# Insulin- and exercise-induced phosphoproteomics of human skeletal muscle identify REPS1 as a regulator of muscle glucose uptake

**DOI:** 10.1016/j.xcrm.2025.102163

**Published:** 2025-06-06

**Authors:** Jeppe Kjærgaard, Cecilie B. Lindqvist, Júlia Prats Quesada, Søren Jessen, Farina Schlabs, Amy M. Ehrlich, Caio Y. Yonamine, Mario García-Ureña, Johann H. Schmalbruch, Lewin Small, Martin Thomassen, Anders Krogh Lemminger, Kasper Eibye, Alba Gonzalez-Franquesa, Jacob V. Stidsen, Kurt Højlund, Juleen R. Zierath, Tuomas O. Kilpeläinen, Jens Bangsbo, Jonas T. Treebak, Morten Hostrup, Atul S. Deshmukh

**Affiliations:** 1 Novo Nordisk Foundation Center for Basic Metabolic Research, Faculty of Health and Medical Sciences, University of Copenhagen, Copenhagen, Denmark; 2 The August Krogh Section for Human Physiology, Department of Nutrition, Exercise and Sports, University of Copenhagen, Copenhagen, Denmark; 3 Steno Diabetes Center Odense, Odense University Hospital, Odense C, Denmark; 4 Department of Clinical Research, University of Southern Denmark, Odense C, Denmark; 5 Department of Physiology and Pharmacology, Integrative Physiology, Karolinska Institutet, Stockholm, Sweden; 6 Department of Molecular Medicine and Surgery, Integrative Physiology, Karolinska Institutet, Stockholm, Sweden

**Keywords:** skeletal muscle signaling, phosphoproteomics, glucose metabolism, insulin, exercise, REPS1, RSK

## Abstract

Skeletal muscle glucose uptake, essential for metabolic health, is regulated by both insulin and exercise. Using phosphoproteomics, we analyze skeletal muscle from healthy individuals following acute exercise or insulin stimulation, generating a valuable dataset. We identify 71 phosphosites on 55 proteins regulated by both stimuli in the same direction, suggesting a convergence of exercise and insulin signaling pathways. Among these, the vesicle-associated protein, REPS1, is highly phosphorylated at Ser709 in response to both stimuli. We identify p90 ribosomal S6 kinase (RSK) to be a key upstream kinase of REPS1 S709 phosphorylation and that the RSK-REPS1 signaling axis is involved in insulin-stimulated glucose uptake. Insulin-induced REPS1 Ser709 phosphorylation is closely linked to muscle and whole-body insulin sensitivity and is impaired in insulin-resistant mice and humans. These findings highlight REPS1 as a convergence point for insulin and exercise signaling, presenting a potential therapeutic target for treating individuals with insulin resistance.

## Highlights

• Comprehensive map of insulin- and exercise-regulated phosphoproteome in human skeletal muscle
• REPS1 S709 is phosphorylated by insulin and exercise via p90 ribosomal S6 kinase
• REPS1 S709 phosphorylation correlates with insulin sensitivity in humans and rodents
• REPS1 phosphorylation is involved in glucose uptake in skeletal muscle

## Graphical abstract

**Figure:**
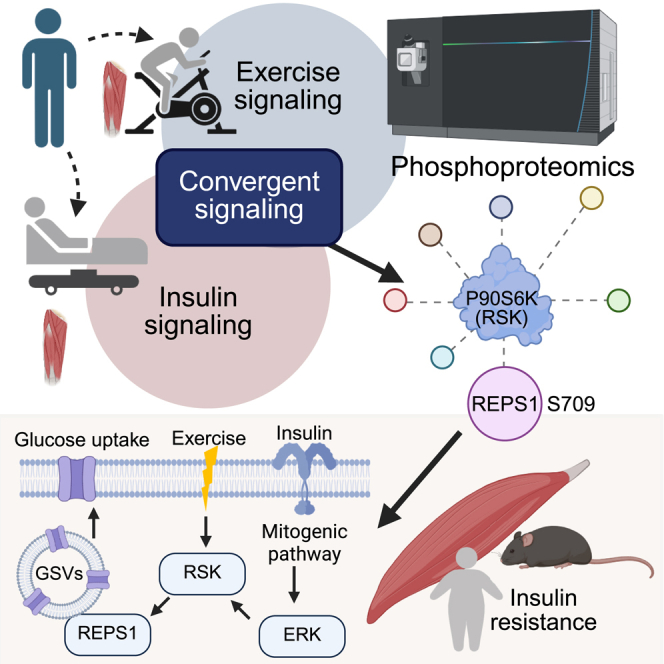

## Teaser

Kjærgaard et al. present a comprehensive map of insulin- and exercise-regulated phosphoproteome in human skeletal muscle. They identify REPS1 S709 being phosphorylated by both stimuli via RSK and involved in glucose uptake. REPS1 phosphorylation correlates with insulin sensitivity and is impaired in insulin resistance, highlighting its role in metabolic regulation.

## Introduction

Skeletal muscle is the largest tissue in the human body and plays a pivotal role in maintaining glucose homeostasis and energy metabolism. One of its primary metabolic functions is glucose uptake and utilization, which is essential for normal physiological processes and is particularly relevant in the context of metabolic disorders, such as type 2 diabetes (T2D).^1^ Impaired insulin-stimulated skeletal muscle glucose uptake is a hallmark of insulin resistance and is a central feature of the pathogenesis of T2D.^2^ Skeletal muscle glucose uptake is intricately regulated by multiple signaling pathways that dictate its delivery, transport, and metabolism.^3^^,^^4^^,^^5^ Glucose transporter 4 (GLUT4) plays a central role in glucose uptake.^6^^,^^7^ During unstimulated conditions, GLUT4 predominantly resides within intracellular vesicles. However, in response to insulin or exercise, GLUT4-containing vesicles translocate to the plasma membrane.^5^ This suggests that exercise- and insulin-induced GLUT4 translocation rely on commonly regulated distal mechanisms and protein machinery. However, the specific signal transducers through which insulin and exercise mediate glucose uptake represent a critical knowledge gap. Identifying these molecular regulators is essential for developing targeted strategies to enhance glucose uptake, which could have significant therapeutic potential for people with insulin resistance and T2D.

Studies on human skeletal muscle in people with T2D have revealed impairment in insulin signaling pathways targeting GLUT4, while exercise-mediated signals remain largely intact.^8^^,^^9^^,^^10^ The study of insulin signaling has mainly been limited to two major pathways: phosphatidylinositol 3-kinase (PI3K)-AKT and RAC1-p21-activated kinase (PAK) pathways, which are recognized as vital modulators of glucose transport.^11^^,^^12^^,^^13^ In contrast, exercise-mediated glucose uptake is mainly orchestrated through signaling mechanisms integrating a spectrum of mechanical, chemical, and stress signals.^14^^,^^15^^,^^16^^,^^17^^,^^18^ One hypothesis is that insulin- and exercise-triggered signaling converge at distal nodes to meet common cellular needs, including glucose import. This notion was supported by the discovery of the molecular switches, TBC1D1 and TBC1D4, which intricately regulate the action of insulin and exercise on the intracellular trafficking of GLUT4 and lipid transporter-containing vesicles to the cell surface.^19^^,^^20^^,^^21^^,^^22^ Intriguingly, an acute bout of exercise can potentiate subsequent insulin signaling, notably by the AMP-activated protein kinase (AMPK)-TBC1D4 axis in both human and rodent skeletal muscle.^23^^,^^24^^,^^25^ Despite these few examples, the understanding of these converging distal mechanisms and the shared protein machinery remains incomplete and underexplored.

Recent improvements in mass spectrometry (MS)-based phosphoproteomics have advanced the study of dynamic protein signaling.^26^^,^^27^^,^^28^ Pioneering studies in skeletal muscle have unveiled extensive signaling networks activated by both exercise and insulin.^29^^,^^30^^,^^31^ Despite these valuable insights, the intricate interplay among these pathways and their connection to GLUT4 remains to be fully elucidated. A comprehensive understanding of these interactions is crucial for unraveling therapeutic targets that can increase skeletal muscle glucose uptake in diseases characterized by insulin resistance. Based on the current evidence, we hypothesized that a core set of proteins, modulated by phosphorylation in response to both insulin and exercise, form a fundamental regulatory nexus for skeletal muscle glucose uptake.

We conducted a phosphoproteomic analysis in skeletal muscle from healthy individuals in response to an acute bout of exercise or insulin stimulation by a hyperinsulinemic euglycemic clamp (HEC). While the majority of phosphosites regulated by both insulin and exercise displayed opposite responses to the stimulus, we identified 71 sites on 55 proteins that were regulated in the same direction, potentially influencing glucose uptake. Notably, we found that RalBP1-associated Eps domain-containing protein 1 (REPS1), a vesicle-associated protein, was phosphorylated in response to both insulin and exercise. We provide evidence that p90 ribosomal S6 kinase (RSK) is a major upstream kinase of REPS1 S709 phosphorylation, and this signaling axis is crucial for insulin- and contraction-stimulated glucose uptake in skeletal muscle. Moreover, insulin action on REPS1 S709 phosphorylation was impaired in several *in vivo* models of insulin resistance and strongly correlated with insulin sensitivity. Our results position REPS1 at the intersection of insulin and exercise signaling, highlighting its key role in glucose uptake. Additionally, the phosphoproteomics dataset offers a valuable resource for dissecting the molecular mechanisms governing skeletal muscle glucose uptake and may aid in the identification of potential targets for therapeutic intervention in people with insulin resistance and T2D.

## Results

### Phosphoproteomic signature of insulin and exercise signaling in human skeletal muscle

**Figure 1:**
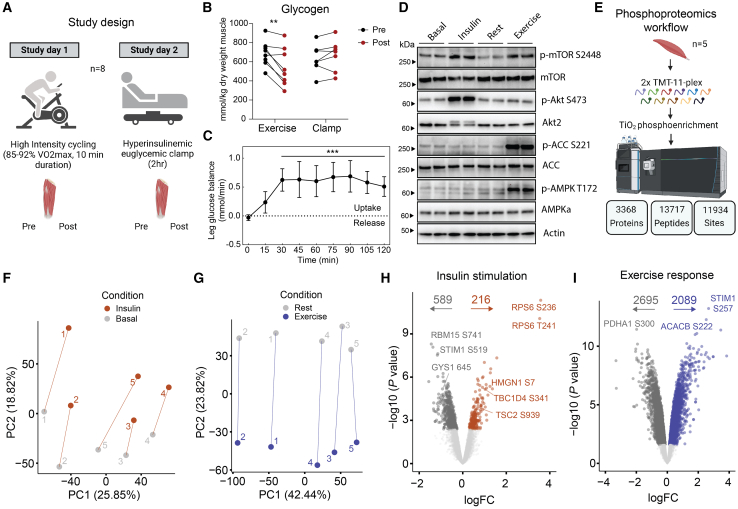
Phosphoproteomic signature of insulin and exercise signaling in human skeletal muscle (A) Study design: eight healthy men underwent, in randomized order, an acute bout of high-intensity cycling exercise (study day 1) and a hyperinsulinemic euglycemic clamp (study day 2). Skeletal muscle biopsies were obtained before and immediately after each intervention. (B and C) Glycogen content (B) and leg glucose balance during clamp (C). (D) Western blot confirmation of insulin and exercise-induced signaling. (E) Phosphoproteomics workflow with 2x TMT 11-plex labeling, phosphopeptide enrichment, and LC-MS/MS analysis. (F and G) Principal-component analysis of insulin and exercise signaling responses. (H–I) Volcano plots of insulin and exercise signaling (*x* axis = logFC, *y* axis = −log10 (*p* value)). A linear model (limma) was used to test for difference in phosphosite abundance between conditions with a false discovery rate set to 5%. Mean +/− standard deviation is represented in (C). *N* = 8 individuals are presented in (A)–(D). *N* = 5 individuals are presented in (E)–(I). Two-sided paired t test was used to test for mean differences in (B). ∗∗*p* < 0.01, ∗∗∗*p* < 0.001.

To accurately capture signaling events in response to insulin and exercise, we conducted two experimental trials, in randomized order, separated by 1–2 weeks on eight healthy males (age, 18–36 years, lean mass index, 14–22 kg/m^2^; maximal oxygen uptake [VO_2max_], 40–60 mL/kg/min). Given our objective to identify converging distal signaling events in response to insulin and exercise, we conducted both trials on the same individuals to minimize potential inter-subject variations in insulin and exercise signaling. During the experimental trials, participants underwent either a 2-h HEC or performed a single bout of high-intensity cycling exercise for 10 min at maximal effort (Figure 1A). We collected vastus lateralis muscle biopsies immediately before and after HEC or exercise on the experimental days. The acute bout of exercise reduced glycogen stores (*p* < 0.01), while no change was observed with insulin stimulation (Figure 1B). The levels of plasma epinephrine and norepinephrine rose during exercise but were unchanged during the clamp procedure (Figures S1A–S1D). Conversely, plasma insulin levels were only increased during the clamp but unchanged during the acute bout of exercise (Figures S1E and S1F). Insulin stimulation increased leg glucose uptake (*p* < 0.001, Figure 1C). We confirmed the activation of canonical insulin and exercise-induced signaling pathways in the muscle biopsies by western blot analysis (Figure 1D). For instance, both insulin and acute exercise induced phosphorylation of mechanistic target of rapamycin (mTOR) at S2448, while insulin specifically increased p-AKT at S473 and exercise specifically increased p-AMPK at T172 and p-acetyl-CoA carboxylase (ACC) at S221 (Figures 1D and S1G–S1J).

Subsequently, we performed in-depth analysis of the phosphoproteome in insulin- and exercise-stimulated skeletal muscle. Proteomic analysis of skeletal muscle is inherently challenging due to the presence of highly abundant contractile proteins hindering the detection of lowly abundant proteins.^32^ To overcome this, we employed stable isotope labeling with 11-plex tandem mass tag (TMT), phosphopeptide enrichment, and phosphopeptide-level fractionation prior to liquid chromatography- tandem mass spectrometry (LC-MS/MS). This approach allowed a comprehensive profiling of human skeletal muscle phosphoproteome, quantifying 13,717 phosphopeptides (11,934 phosphosites localization probability >0.75) on 3,368 proteins (Figure 1E). A principal-component analysis demonstrated the clustering of samples by subject on component 1 and by stimulus on component 2 (Figures 1F and 1G). Differential abundance analysis of the phosphoproteome revealed significant regulation with 805 and 4,784 sites changing in response to insulin and exercise, respectively (false discovery rate (FDR) < 0.05, Figures 1H and 1I). This included known insulin- (upregulation of RPS6 S236/T241, TBC1D4 S341, and TSC2 S939 and downregulation of GYS1 S645) and exercise-signaling proteins (upregulation of STIM1 S257 and ACACB S222 and downregulation of PDHA1 S300) as well as additional insulin-signaling proteins, such as the S741 phosphosite on the RNA-binding protein 15, RBM15, which was downregulated in response to insulin. Interestingly, RBM15 was recently associated with liver insulin resistance through epigenetic regulation.^33^ Our comprehensive phosphoproteomic analysis encompassing insulin and exercise stimuli in the same individuals represents a valuable resource for the scientific community (Tables S1A–S1C).

### Shared and distinct features of insulin and exercise signaling

**Figure 2:**
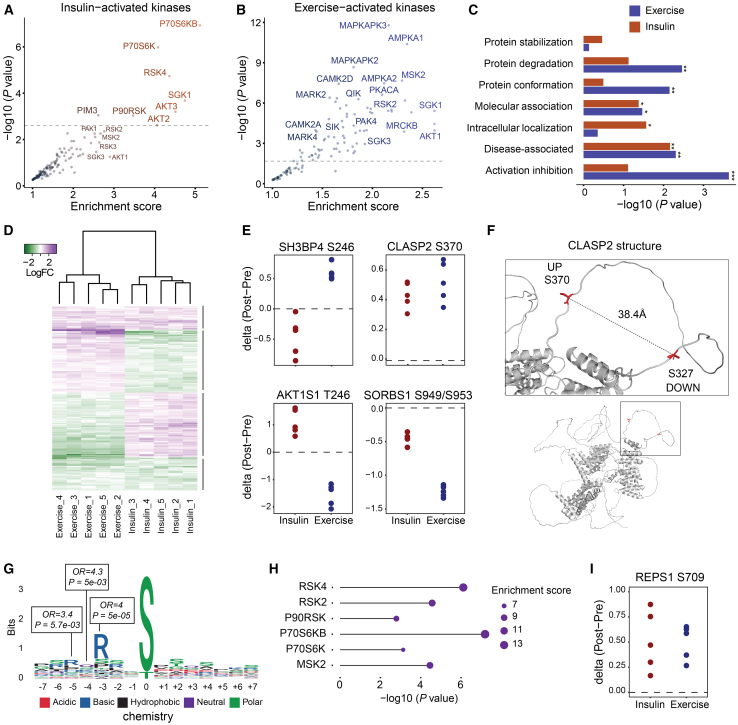
Shared and distinct features of insulin and exercise signaling Predicted insulin- and exercise-activated kinases from the kinase library (adjusted *p* value < 0.05) (A and B). Two-sided Fisher’s exact test of phosphosite functionality based on the PhosphoSitePlus database (C). Heatmap of individual fold-change response of significant phosphosites to exercise and insulin stimulation across the five subjects (D). Selected phosphosites regulated in opposite or the same direction (E). AlphaFold-predicted structure of CLASP2 with highlighted serine residues phosphorylated or dephosphorylated by both insulin and exercise (F). Sequence window of phosphosites upregulated by insulin and exercise (G). A two-sided Fisher’s exact test was used to test for residue overrepresentation. Kinase enrichment analysis of phosphosites upregulated by both insulin and exercise (H). Individual fold changes of p-REPS1 S709 in response to insulin and exercise (I). ∗*p* < 0.05, ∗∗*p* < 0.01, ∗∗∗*p* < 0.001.

To decipher the signaling pathways and regulatory networks activated by insulin and exercise, we investigated if specific kinases were activated and potentially responsible for the observed phosphorylation events in our dataset. Recent work on mapping substrates of the human Ser/Thr kinome has provided *in vitro* evidence of kinase-substrate relationship.^34^ Leveraging this work, we overlaid our sequence windows surrounding phosphorylated serine and threonine residues with the *in vitro*-predicted kinase motifs. As substrates are essential for interaction with their upstream kinase, we filtered for kinases known to be expressed in skeletal muscle tissue.^35^ Subsequently, we performed a kinase enrichment analysis, predicting the activation of known kinases (P70S6K, P90S6K/RSK, and SGK1) and a hitherto previously unrecognized insulin-activated kinase, PIM3, in response to insulin (Figure 2A and Table S1E). Exercise resulted in the activation of known kinases such as PKACA, AMPKα1, AMPKα2, MAPKAPK2, MAPKAPK3, and RSK2 (Figure 2B and Table S1F) and previously uncharacterized kinases such as QIK (also known as salt-inducible kinase 2,). Our dataset, highlighting previously unrecognized kinases activated by insulin and exercise serves as a valuable resource for exploring their role in skeletal muscle function and metabolism. Phosphosites regulated by exercise were enriched for processes related to protein degradation, protein conformation, and protein activation/inhibition while those regulated by insulin were associated with intracellular localization (two-sided fishers exact test *p* < 0.05) (Figure 2C). Furthermore, sites with a reported function in molecular associations (i.e., interactions) or disease-associated function were enriched by both insulin and exercise (Figure 2C).

To elucidate the crosstalk between insulin and exercise-induced signaling, we filtered for significantly altered sites by both insulin and exercise (<5% FDR in both comparisons). In total, this analysis revealed 233 phosphosites that were regulated in either the same or opposite direction (Figure 2D and Tables S1C and S1D). When examining individual responses to exercise and insulin, we observed that the majority of phosphosites, 162 in total, were regulated in opposite directions, compared to 71 sites regulated in the same direction. This suggests that insulin and exercise induce distinct and opposing signaling events, which is in line with their well-established roles in promoting anabolic and catabolic metabolism, respectively. One example is the known T246 site of AKT1S1 (also known as PRAS40), which was phosphorylated in response to insulin stimulation and dephosphorylated on the same site in response to exercise (Figure 2E). Conversely, SH3BP4, a protein involved in autophagy and receptor endocytosis, displayed dephosphorylation on S246 in response to insulin and phosphorylation in response to exercise.^36^ Exercise and insulin are known to induce and attenuate autophagy in skeletal muscle, respectively.^37^^,^^38^ Therefore, the S246 phosphorylation on SH3BP4 could be a key regulatory step in skeletal muscle autophagy.

We hypothesized that the phosphosites regulated in the same direction during insulin and exercise could be involved in metabolic processes such as skeletal muscle glucose uptake. Accordingly, these sites were enriched for biological processes related to exocytosis, actin filament organization, and endomembrane system organization (Figure S2A). Similarly, sites downregulated with both stimuli were enriched for cytoskeletal organization (Figure S2B). Exploring specific examples, we observed that SORBS1 (also known as Cbl-associated protein) was dephosphorylated on both S949 and S953 (same phosphopeptide) in response to both insulin and exercise (Figure 2E). SORBS1 is required for insulin-stimulated glucose uptake in adipocytes independent of the canonical PI3K pathway.^39^ However, its role in skeletal muscle insulin signaling has been debated. Additionally, we observed that cytoplasmic linker-associating protein (CLASP)-2 S370 phosphorylation was induced in response to both insulin and exercise (Figure 2E), while CLASP2 S327 was dephosphorylated in response to both stimuli (Figure S2C). When we visualized the AlphaFold-predicted structure, we found both sites to be present in the same loop domain suggesting interdependency (Figure 2F). The same observation was seen on the sister protein CLASP1 at S600 and S559 (Figures S2D and S2E). CLASP2 is required for insulin-stimulated GLUT4 translocation, thereby serving as a positive readout for our phosphoproteomics analysis.^40^^,^^41^ In total, we identified 29 phosphosites corresponding 27 proteins induced by both insulin and exercise. To gain further insights into their upstream kinases, we conducted a motif enrichment analysis, revealing a prevalent sequence motif surrounding the phosphosites with a highly significant proportion of arginine at position −3 (odds ratio = 4, *p* = 5e−05) (Figure 2G). Additionally, these motifs were enriched for P70S6K and P90S6K (RSKs) kinases (Figure 2H). Interestingly, prior research has shown that S6K and RSK are activated by both insulin and exercise in human skeletal muscle.^42^^,^^43^^,^^44^^,^^45^ In addition, RSK1 was recently found to induce GLUT4 translocation in adipocytes through TBC1D4 phosphorylation in response to stress stimuli.^46^ Of particular interest, we identified the protein REPS1 to be phosphorylated on S709 in response to insulin and exercise (Figure 2I).

### The insulin- and exercise-responsive protein, REPS1, is involved in skeletal muscle glucose uptake

**Figure 3:**
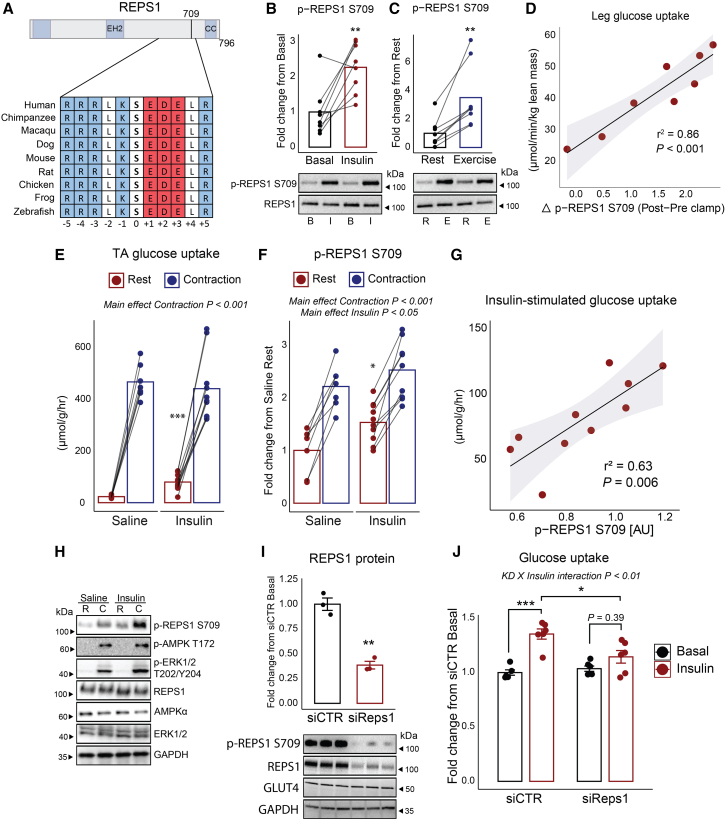
The insulin and exercise-responsive protein, REPS1, is a critical regulator of skeletal muscle glucose uptake Highly conserved REPS1 sequence window around the serine 709 site (A). Western blot validation of REPS1 phosphorylation in human skeletal muscle before (basal) and after (insulin) a 2-h hyperinsulinemic euglycemic clamp (B) and at rest and after 10 min high-intensity cycling exercise (C). Pearson’s correlation analysis of steady-state leg glucose uptake and delta (post-pre clamp) REPS1 S709 phosphorylation (D). *In vivo*^3^H-2-deoxyglucose uptake during rest or *in situ* contractions of tibialis anterior (TA) muscle with saline or 15 mU insulin stimulation (E). Western blot analysis of REPS1 phosphorylation during rest or *in situ* contractions of TA muscle with saline or 15 mU insulin stimulation (F). Pearson’s correlation analysis of insulin-stimulated glucose uptake into TA muscle and REPS1 S709 phosphorylation (G). Representative western blot from *in situ* contraction experiment (H). Western blot validation of siRNA-mediated knockdown of *Reps1* in C2C12 myotubes (I). Insulin-stimulated ^3^H-2-deoxy-glucose uptake in C2C12 myotubes transfected with control or *Reps1* siRNA (J). Data in (B) and (C) were analyzed with a two-sided paired-sample t test (*N* = 8). Data in (E) and (F) were analyzed by a two-way ANOVA with repeated measures and Sidak multiple comparison test (*n* = 7–10). Data in (I) were analyzed by a two-sided two-sample t test. Data in (J) were analyzed by two-away ANOVA with Tukey’s multiple comparisons test (*n* = 6) (J). In paired analyses, bars represent the mean. In unpaired analyses, the mean +/− standard error of the mean is displayed. ∗*p* < 0.05, ∗∗*p* < 0.01, ∗∗∗*p* < 0.001.

In our search for previously unidentified signal transducers of glucose uptake, we identified that REPS1 S709 was phosphorylated in response to both insulin and exercise stimuli. The amino acid sequence surrounding serine 709 was found to be conserved from zebrafish to humans containing the motif favored by AGC family kinases^47^^,^^48^ (Figure 3A). Moreover, REPS1 has been characterized for its involvement in endocytosis.^47^ Therefore, we prioritized REPS1 for further studies to validate its role in mediating glucose uptake in skeletal muscle. We validated our observation by immunoblotting for phospho-REPS1 at S709 in human skeletal muscle, confirming an increase in phosphorylated REPS1 at S709 in response to both insulin and exercise (Figures 3B and 3C). Strikingly, REPS1 S709 phosphorylation (post-pre clamp) correlated tightly with insulin-stimulated steady-state leg glucose uptake with a squared Pearson’s correlation coefficient of 0.86 (*p* < 0.001) (Figure 3D). In contrast, p-AKT S473 and p-mTOR S2448, classical markers of insulin signaling, showed poor correlation (Figures S3A and S3B). This may be explained by the “sparseness” hypothesis, where minimal Akt activation suffices for glucose uptake,^49^ differences in timing, as these phosphorylation events may peak at earlier time points and decline by the time glucose uptake is measured, contributing to the weak correlation observed. To assess the association of REPS1 S709 phosphorylation with glucose uptake during exercise in human skeletal muscle *in vivo*, we examined REPS1 S709 phosphorylation in muscle biopsies obtained prior to and after a one-legged exercise and compared this with corresponding femoral arterial-venous plasma glucose balance. REPS1 S709 phosphorylation displayed a small but significant (*p* < 0.05) increase immediately after 10 min of exercise (Figures S3C and S3E). However, the REPS1 phosphorylation was not associated with glucose uptake in the exercised leg (r^2^ = 0.01, *p* = 0.85) (Figure S3D). This suggests that REPS1 709 phosphorylation is closely associated with skeletal muscle insulin-stimulated glucose uptake but does not reflect the amount of glucose uptake during exercise.

To ascertain the conservation of response, we anesthetized C57BL/6NTac male mice and electrically stimulated one leg to contract while the contralateral leg remained rested. Upon contractions, mice were injected with either saline or 15 mU insulin combined with ^3^H-2-deoxyglucose for 15 min. Tibialis anterior (TA) muscle had a ∼20-fold and 3.4-fold increase in glucose uptake during contractions and insulin, respectively; however, no additive effect was observed (Figure 3E). We also measured REPS1 S709 phosphorylation and found increased phosphorylation in all contracted muscles in response to 15 min of *in situ* contraction (main effect *p* < 0.001) (Figures 3F and 3H). As observed in humans, 15 min of *in vivo* insulin stimulation increased REPS1 S709 phosphorylation (main effect *p* < 0.05). The insulin-stimulated glucose uptake into TA muscle had a strong correlation with REPS1 S709 phosphorylation (r^2^ = 0.63, *p* = 0.0063) (Figure 3G) whereas contraction-stimulated glucose uptake showed poor correlation (r^2^ = −0.064, *p* = 0.58) (Figure S3F). To test if REPS1 is required for insulin-stimulated glucose uptake, we transfected C2C12 muscle cells with small interfering RNA (siRNA) against mouse *Reps1*. Efficient knockdown of *Reps1* was validated at the protein level by western blot analysis (*p* < 0.01), without any changes in GLUT4 expression (Figure 3I). Insulin-stimulated ^3^H-2-deoxyglucose was impaired when REPS1 protein expression was reduced (interaction *p* < 0.01) (Figure 3J). Collectively, our results show that insulin and exercise induce REPS1 S709 phosphorylation, with RESP1 being required for insulin-stimulated glucose uptake.

### RSK is an upstream kinase of REPS1 S709 and is required for insulin-stimulated GLUT4 translocation and glucose uptake

**Figure 4:**
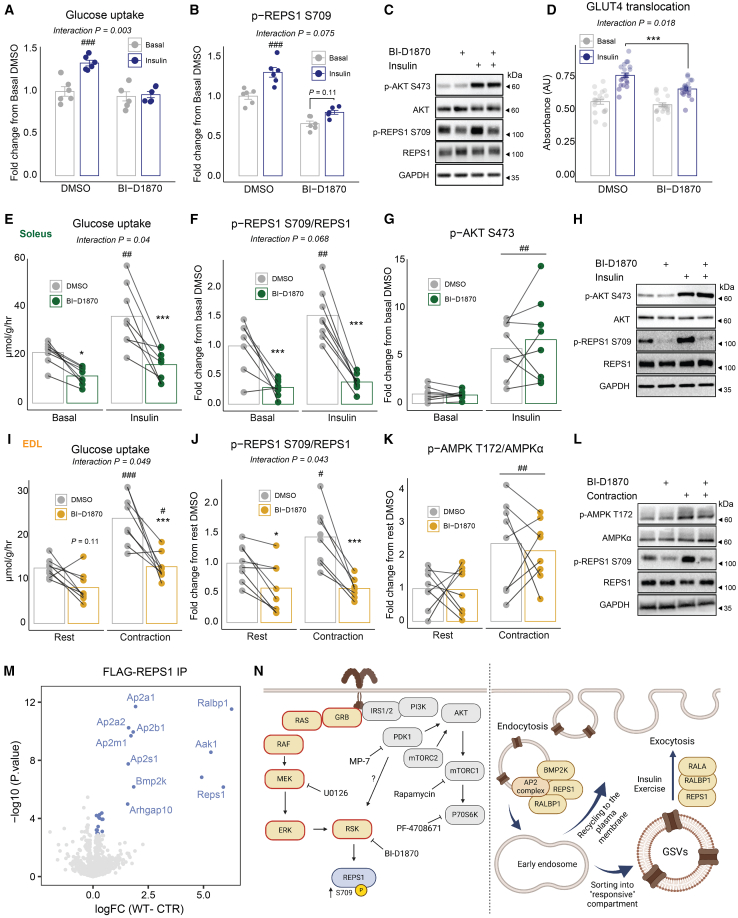
RSK is an upstream kinase of REPS1 S709 and is associated with vesicle-sorting proteins in skeletal muscle (A) Insulin-stimulated (100 nM, 10 min) glucose uptake in C2C12 myotubes preincubated (20 min) with DMSO or 10 μM of the RSK inhibitor, BI-D1870. (B) Quantified western blot analysis of signaling as in (A). (C) Representative western blot analysis for signaling as in (B). (D) Insulin-stimulated (100 nM, 10 min) GLUT4 translocation in L6 myotubes preincubated (20 min) with DMSO or 10 μM BI-D1870. (E) Basal and insulin-stimulated (300 μIU/mL, 30 min) glucose uptake in *ex vivo*-incubated mouse soleus muscle preincubated (60 min) with DMSO or 10 μM BI-D1870. (F and G) Quantified western blot analysis of signaling as in (E). (H) Representative images of western blot analysis for signaling in *ex vivo* insulin-stimulated muscles. (I) Rested and contraction-stimulated glucose uptake in *ex vivo*-incubated mouse extensor digitorum longus (EDL) muscle preincubated (60 min) with DMSO or 10 μM BI-D1870. (J and K) Quantified western blot analysis of signaling as in (I). (L) Representative images of western blot analysis for signaling in *ex vivo* contraction-stimulated muscles. (M) FLAG pull-down in C2C12 myotubes transduced with either FLAG-*Reps1* or FLAG-CTR . (N) Differentially enriched proteins highlighted in blue (two-sample t test, FDR <5%). Illustration of signaling pathways affected and the proposed role of REPS1 in vesicle trafficking. The illustration was made in BioRender. ∗symbol refers to significance of inhibitor vs. DMSO treatment. # symbol refers to significance of insulin/contraction. ∗∗∗/###*p* < 0.001, ∗∗/##*p* < 0.01, ∗/#*p* < 0.05. Data in (A), (B), (C), and (D) were analyzed by two-way ANOVA with Tukey’s multiple comparisons test. Data in (E)–(G) and (I)–(K) were analyzed by a two-way ANOVA with repeated measures with Šidák’s multiple comparisons test (*n* = 7–8). In paired analyses, bars represent the mean. In unpaired analyses, the mean +/− standard error of the mean is displayed.

We hypothesized that a single upstream kinase could be activated during insulin and exercise stimulation and be responsible for the increased phosphorylation of REPS1 S709. Interestingly, the sequence motifs of phosphosites upregulated by both insulin and exercise were enriched for the P70S6K and P90S6K isoforms (Figure 2H). In support, a recent study found that RSK can phosphorylate REPS1 at S709 *in vitro* in 293T cells.^50^ To investigate if RSK is involved in insulin-stimulated glucose uptake, we preincubated C2C12 myotubes with the RSK inhibitor, BI-D1870, for 20 min followed by 10 min of maximal insulin stimulation. BI-D1870 is an established highly specific RSK inhibitor without effects on extracellular signal-regulated kinase (ERK), protein kinase A (PKA), PDK1, AKT, S6K, and the RSK-related kinase MSK1.^50^^,^^51^ Glucose uptake and REPS1 S709 phosphorylation increased in response to insulin; however, these effects were blunted upon RSK inhibition. (interaction *p* = 0.003 and *p* = 0.075, respectively) (Figures 4A–4C). Importantly, the RSK inhibitor lowered phosphorylated REPS1 without affecting insulin-stimulated phosphorylation of AKT on S473 (Figure S4A). Furthermore, the basal level of phosphorylated REPS1 on S709 was lowered suggesting that RSK is the upstream kinase. Using an L6-GLUT4-myc model, we next investigated whether REPS1 phosphorylation is required for the translocation of GLUT4 to the cell surface. We confirmed that the reduced insulin-stimulated glucose uptake by blocking RSK-REPS1 signaling was due to an impaired GLUT4 translocation to the plasma membrane (interaction *p* = 0.018, Figure 4D).

To translate these findings into adult intact muscle, we dissected soleus muscle from mice and incubated with or without the addition of a submaximal insulin dose (300 μIU/mL) in the presence or absence of RSK inhibitor (10 μM, 1-h pre-incubation). Notably, RSK inhibition impaired insulin-stimulated glucose uptake (interaction *p* = 0.04, Figure 4E). We also confirmed that REPS1 phosphorylation was impaired in the same muscles (interaction *p* = 0.068, Figure 4F), despite full activation of the AKT pathway (Figures 4G and 4H).

To test whether insulin-induced REPS1 S709 phosphorylation is also dependent on the mTOR-S6K pathway, we stimulated C2C12 myotubes with Rapamycin and PF-4708671, known inhibitors of mTORC1 or P70S6K, respectively. These inhibitors reduced insulin-stimulated phosphorylation of P70S6K T389 (mTOR site) and RPS6 S235/236 (P70S6K site), without affecting REPS1 S709 phosphorylation (Figures S4B–S4D). As RSK can also be activated by 3-phosphoinositide-dependent protein kinase 1 (PDK1), we pre-treated cells with a PDK1 inhibitor, MP-7, but found no impairment in insulin-stimulated phosphorylation of REPS1 S709 despite markedly reduced phosphorylation of the PDK1 site, T308, on AKT (Figures S4E–S4G). RSK is also known to be activated through the RAF-MEK-ERK pathway^52^^,^^53^ (Figure 4N, left). Therefore, we inhibited the mitogen-activated protein kinase (MAPK) pathway and observed concurrent reduction in ERK1/2 T202/Y204 (interaction *p* < 0.001) and REPS1 S709 phosphorylation (interaction *p* = 0.02), indicating that signaling from the insulin receptor to REPS1 S709 is mediated through the MAPK pathway (Figures S4H–S4J).

### The RSK-REPS1 signaling axis is partially required for contraction-stimulated glucose uptake

As REPS1 S709 phosphorylation increases in response to an acute bout of exercise and contractions, we aim to test whether the RSK-REPS1 signaling axis is required for *ex vivo* contraction-induced glucose uptake. We first examined whether REPS1 S709 phosphorylation increased in response to *ex vivo* contractions. We found that contraction-induced REPS1 S709 phosphorylation was exclusive to the extensor digitorum longus (EDL) muscle and absent in the soleus muscle (Figures S5A and S5B). Based on this, we focused on the EDL and preincubated the muscle with the RSK inhibitor prior to and throughout 10 min of *ex vivo* contractions. As expected, in control muscle, contractions significantly increased muscle glucose uptake (Figure 1, *p* < 0.001). RSK inhibition impaired contraction-stimulated glucose uptake by 58% (interaction *p* = 0.049, Figure 4I, S5C, and S5D). Additionally, contraction-stimulated REPS1 S709 phosphorylation was completely blunted confirming that RSK is the major upstream kinase of REPS1 S709 phosphorylation in response to contractions (interaction *p* = 0.043, Figure 4J). Phosphorylated AMPK T172 increased equally in both control and RKS-inhibited muscles, indicating that activation of canonical stress signaling pathways was unaffected (Figures 4K and 4L). Although REPS1 phosphorylation is responsive to both insulin and contraction in certain muscle types, its role in contraction-mediated glucose uptake may be muscle fiber-type dependent. The absence of significant REPS1 phosphorylation in the soleus suggests that contraction-stimulated glucose uptake in oxidative muscle may occur through alternative mechanisms. Future studies exploring fiber-type-specific signaling responses will be necessary to fully delineate the role of REPS1 in contraction-induced glucose uptake.

### REPS1 interacts with proteins involved in endocytotic and exocytotic vesicle dynamics

The S709 residue of REPS1 lies within a disordered region where phosphorylation could trigger gain/loss of protein interactions. To identify REPS1 binding partners, we applied adeno-associated virus-assisted overexpression of FLAG-control (CTR) or FLAG-*Reps1*. We next performed MS-based interaction proteomics. As expected, REPS1 was the most enriched protein in the WT-FLAG vs. CTR-FLAG (Figure 4M). RALBP1, a protein recently shown to bind REPS1 and regulate GLUT4 exocytosis in fibroblast, co-precipitated with REPS1 in our screen.^54^ Furthermore, all five subunits of the endocytotic adaptor protein 2 (AP-2) complex (Ap2a1, Ap2a2, Ap2b1, Ap2m1, and Ap2s1) and associated kinases, Aak1 and Bmp2k, were bound to REPS1. These interactors were similarly found by immunoprecipitation and MS-based proteomics of endogenous REPS1 (Figure S5K). The REPS1 interactors confidently found in both FLAG and endogenous immunoprecipitation were enriched for processes related to endocytosis and vesicle transport (Figures S5E–S5G). In summary, our interactome analysis shows that REPS1 binds to proteins related to both the exocytosis and endocytosis machinery (Figure 4N, right)..

### Insulin-induced REPS1 S709 phosphorylation *in vivo* is impaired in multiple models of insulin resistance

**Figure 5:**
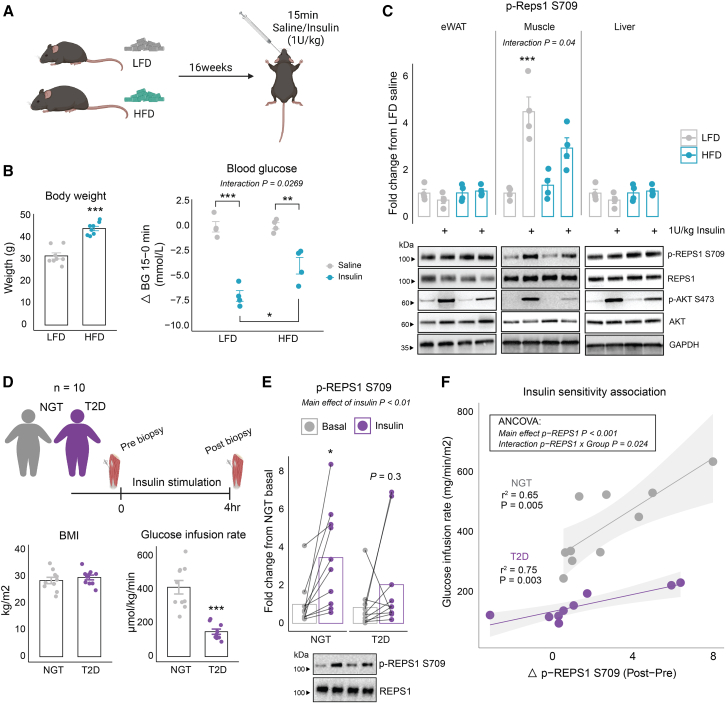
Insulin-induced REPS1 S709 phosphorylation *in vivo* is impaired in multiple models of insulin resistance Sixteen C57BL6 male mice (7 weeks old) were fed a low-fat diet (LFD; *n* = 8) or high-fat diet (HFD; *n* = 8) for 16 weeks. At the day of termination, mice were injected with either saline or 1 U/kg insulin, and tissues were collected after 15 min (*n* = 4) (A). Body weight of mice after 16 weeks of diet intervention and blood glucose (mM) delta (post-pre) values in response to saline/insulin injection (B). Corresponding western blot analysis of insulin signaling in epidydimal white adipose tissue (eWAT), quadriceps muscle, and liver tissue from same animals (C). BMI and glucose-infusion rate (GIR) in response to a hyperinsulinemic euglycemic clamp (4 h) of 10 normal glucose-tolerant (NGT) and 10 patients with T2D (D). Quantified western blot of REPS1 S709 phosphorylation pre- and post-insulin stimulation (E). Association of GIR and delta REPS1 S709 phosphorylation in skeletal muscle (F). Data in (B) and (C) were analyzed by a two-way ANOVA and Tukey’s multiple comparisons test. Data in (E) were analyzed by a two-way ANOVA with repeated measures and Sidak multiple comparisons test. Data in (F) were analyzed by ANCOVA analysis followed by individual Pearson correlation. In paired analyses, bars represent the mean. In unpaired analyses, the mean +/− standard error of the mean is displayed. ∗*p* < 0.05, ∗∗*p* < 0.01, ∗∗∗*p* < 0.001.

After establishing the critical role of REPS1 in skeletal muscle glucose uptake, we sought to investigate whether REPS1 phosphorylation is impaired under insulin-resistant conditions. To elucidate if insulin-stimulated phosphorylation of REPS1 is affected by insulin resistance, we fed C57BL/6NTac male mice a low-fat diet (LFD) or high-fat diet (HFD) for 16 weeks, injected them acutely with saline or insulin (1 U/kg), and tested their blood glucose levels before and 15 min following injection (Figure 5A). Mice fed an HFD had higher body weight (*p* < 0.001) and, upon insulin injection, displayed less reduction in blood glucose compared to LFD-fed mice (interaction *p* < 0.05, Figure 5B), indicating reduced whole-body insulin sensitivity in the HFD group. Intriguingly, the insulin-stimulated phosphorylation of REPS1 at S709 was lower in skeletal muscle of mice fed an HFD compared to those fed an LFD (interaction *p* < 0.05) (Figure 5C). This effect was specific to skeletal muscle, as epididymal white adipose tissue and liver REPS1 phosphorylation were not affected by insulin or diet. This was despite lowered insulin-stimulated AKT S473 phosphorylation in all three tissues. Furthermore, the insulin-induced drop in blood glucose levels correlated with skeletal muscle REPS1 S709 phosphorylation levels (r^2^ = 0.69, *p* = 0.01) (Figure S5H).

To determine if this impairment is present in humans, we analyzed skeletal muscle biopsies from 10 people with T2D and 10 controls matched by age, gender, and BMI with normal glucose-tolerance (NGT) in the resting basal and insulin-stimulated steady-state periods of an HEC (Figure 5D). People with T2D exhibited lower insulin sensitivity than controls (*p* < 0.001) (Figure 5D). REPS1 S709 phosphorylation was increased in response to insulin infusion by 3.5-fold (*p* < 0.05) in the NGT group compared to 2.0-fold in the T2D group (*p* = 0.3) (Figure 5E). We observed a highly heterogeneous response in both groups. Therefore, we correlated the delta (post-pre clamp) response in phosphorylated REPS1 S709 with whole-body insulin sensitivity. Strikingly, there was an interaction in responses between individuals with T2D and NGT (analysis of covariance [ANCOVA] interaction *p* = 0.024) (Figure 5F). Therefore, we tested individual associations within each group and found correlations for both groups (NGT: r^2^ = 0.65, *p* = 0.005, T2D: r^2^ = 0.75, *p* = 0.003) (Figure 5F). Together, these results highlight the role of REPS1 as a key signaling protein impaired in insulin resistance and suggest that enhancing REPS1 phosphorylation could be a potential therapeutic strategy to improve insulin sensitivity and restore glucose uptake in metabolic disorders such as T2D.

### *REPS1* variants’ association with complex traits, mRNA expression, and splicing

**Figure 6:**
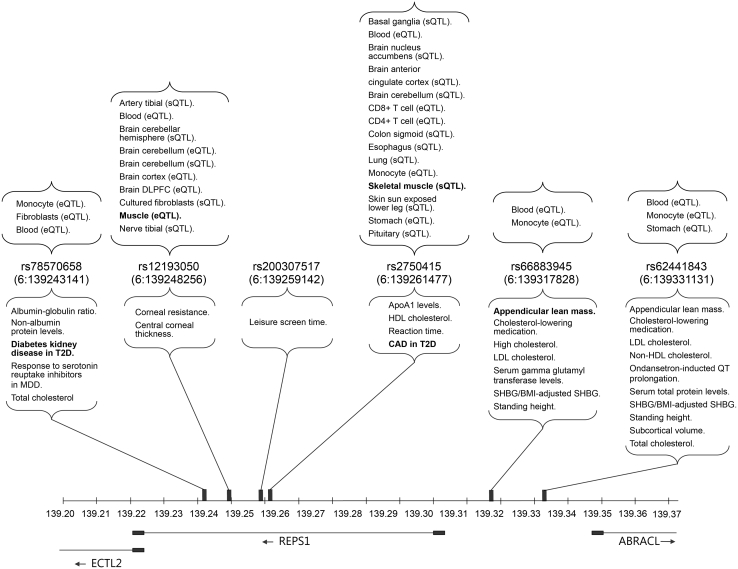
REPS1 variants’ associations with complex traits, mRNA expression, and splicing Lower segment: representation of the phenotypes associated with *REPS1* variants using data from NHGRI-EBI GWAS Catalog and Open Targets Genetics. In total, we identified 6 independent signals (r^2^ < 0.1) in the *REPS1* region: 4 were located in *REPS1* introns and 2 in an intergenic region between *REPS1* and *ABRACL*. Each signal is represented by their lead variant. Upper segment: representation of tissues and cell types with significant changes in *REPS1* expression quantitative trait locus (eQTL) or alternative splicing (sQTL) as reported in Open Targets Genetics. We report all tissues and cell types with significant eQTL or sQTL associations for any of the 6 independent *REPS1* lead variants. See Tables S2A–S2C for more information on the associations for each lead variant.

After identifying that REPS1 was closely linked to glucose uptake and insulin sensitivity, we explored whether *REPS1* gene variants are associated with cardiometabolic health. By analyzing the NHGRI-EBI genome-wide association study (GWAS) catalog, we identified 17 *REPS1* variants linked to 22 distinct phenotypes (*P* < 1 × 10^−6^).^55^^,^^56^^,^^57^^,^^58^^,^^59^ Using the 1000G European reference panel for linkage disequilibrium pruning, we discerned 6 independent *REPS1* signals (r^2^ < 0.1). Employing Open Targets Genetics, we focused on the lead variant with the most significant associations within each signal (Figure 6; Tables S2A and S2B). Remarkably, 4 out of these 6 independent lead variants (rs7870658, rs2750415, rs66883945, and rs62441843) are associated with lipid traits. Two lead variants (rs66883945 and rs62441843), located between *REPS1* and *ABRACL*, also share associations with appendicular lean mass, standing height, and sex-hormone binding globulin. Intriguingly, two lead variants within *REPS1* introns (rs7870658 and rs2750415) are associated with cardiometabolic diseases in T2D, such as coronary artery disease and chronic kidney disease. Finally, two other lead variants in *REPS1* introns are singularly associated with corneal-related traits (rs12193050) and leisure screen time (rs200307517).

To evaluate the association of the 6 independent *REPS1* lead variants with differential expression quantitative trait locus (eQTL) and alternative splicing (sQTL) of *REPS1* across tissues and cell types, we queried each variant in Open Targets Genetics (Figure 6 and Table S2C). The majority of the associations were linked to altered expression in blood (5/5 lead variants), monocytes (4/5 variants), brain (2/5 variants), and fibroblasts (1/5 variants). However, two lead variants displayed association to muscle tissue: rs12193050 acts as an eQTL for muscle, and rs2750415 acts as an sQTL for skeletal muscle. This suggests a potential mechanism in which these genetic variants influence muscle-specific gene regulation of *REPS1*.

## Discussion

Insulin and exercise potently stimulate glucose uptake in skeletal muscle, but the signaling pathways remain largely understudied in clinical material. Here, we mapped the insulin- and exercise-stimulated phosphoproteome of skeletal muscle from the same individuals. Our analysis uncovered both overlapping and distinct signaling events triggered by insulin and exercise in humans, providing a valuable resource for future hypothesis generation and mechanistic studies. REPS1 emerged as a key protein phosphorylated at S709 in response to both exercise and insulin, impacting skeletal muscle glucose uptake. Moreover, phosphorylation of REPS1 strongly correlated with insulin sensitivity and was specifically impaired in skeletal muscle under insulin-resistant conditions, emphasizing its potential relevance in metabolic diseases such as T2D.

Insulin and exercise induced substantial changes in protein phosphorylation, particularly exercise, which affected 50% of the phosphoproteome. While most of these changes are consistent with previous phosphoproteomic studies,^29^^,^^30^^,^^31^ this study presents comprehensive map of the deep phosphoproteome in response to acute insulin and exercise stimulation within the same individuals. The study design minimized inter-subject variability and allowed for a precise interrogation of both distinct and shared signaling events induced by these diverse stimuli. This dataset has the potential to serve as a valuable resource for generating innovative hypotheses. From our analysis, 71 phosphosites consistently responded to both insulin and exercise, of which some are potentially involved in modulating glucose uptake. We highlight an intriguing (de)phosphorylation event on the protein CLASP2, where both insulin and exercise led to dephosphorylation of the S327 residue and phosphorylation of the S370 residue, close in space in a disordered region. This mechanism was also observed on the sister protein CLASP1 (site S559 and S600). CLASP2 regulates insulin-stimulated microtubule dynamics, is directly associated with GLUT4, and is required for insulin-stimulated glucose uptake.^40^^,^^41^ Therefore, CLASP1/2 are likely core proteins required for exercise/insulin-stimulated GLUT4 translocation. Additionally, we also identified that vesicle-associated membrane protein-associated protein A (VAPA, also known as VAP-33) was phosphorylated on S214 in response to insulin and exercise. VAPA associates with vesicle-associated membrane protein 2 and regulates an insulin-dependent insertion of GLUT4 into the plasma membrane.^60^ Thus, our data indicate that insulin and exercise stimulate an overlapping set of proteins important for translocation and insertion of GLUT4 vesicles into the plasma membrane.

Our major discovery centers on the phosphorylation of REPS1 on S709 in response to insulin and exercise and the investigation of this signal transducer in skeletal muscle glucose uptake. REPS1 was first characterized as a regulator of endocytosis and epidermal growth factor receptor (EGFR) internalization.^47^^,^^48^ However, in HeLa cells, REPS1 knockout does not affect EGFR internalization. Instead transferrin receptor (TfR) recycling is impaired when REPS1 lacks a phosphorylatable S709 site, emphasizing the functional importance of this residue.^61^ Considering GLUT4’s presence in TfR-positive vesicles, which are responsive to insulin,^6^ we propose that REPS1 plays a role in regulating GLUT4 recycling. Our evidence indicates that REPS1 binds to the AP-2 complex and associated kinases (BMP2K and AAK1), suggesting a role in endocytosis, although the specific stage of REPS1 interaction with the endocytic machinery remains unknown. REPS1 forms a complex with RALBP1 in fibroblasts regulating exocytotic processes including trafficking of GLUT4.^54^ As we observed a substantial co-enrichment of RALBP1 with REPS1, we interpret this as further evidence for a role in glucose transport via GLUT4 exocytosis in skeletal muscle. Taken together, we propose a dual role for REPS1: (1) sorting of GLUT4 vesicles in early endosome during endocytosis and (2) acute exocytosis processes possibly through interaction with RALBP1/RALA.

Multiple kinases have been implicated in exercise- and insulin-stimulated glucose uptake in skeletal muscle. Here we identify that phosphosites induced by both insulin and exercise are predicted S6K and RSK kinase family substrates. In addition, acute inhibition of RSK blocked insulin-stimulated glucose uptake and impaired contraction-induced glucose uptake in skeletal muscle cells and tissue. However, REPS1 S709 phosphorylation increased only in glycolytic muscle (EDL) and not in the oxidative soleus muscle. This suggests that REPS1 plays a fiber-type-specific role in contraction-stimulated glucose uptake, with alternative pathways likely contributing in oxidative muscle. Notably, *ex vivo* contraction-stimulated glucose uptake is much higher in EDL than in soleus muscle,^62^ which may further explain the differential phosphorylation response. The role of RSK in glucose uptake is also supported by direct interaction and phosphorylation of TBC1D4 with RSK in adipocytes.^46^ Here RSK1 was required for stress-stimulated glucose uptake. Our findings corroborate a similar axis involving RSK, as we identify RSK as an upstream kinase of REPS1. RSK activation can occur through multiple pathways including the MAPK pathway and by direct phosphorylation by PDK1.^63^ In our study, we did not find evidence that PDK1 or the AKT-mTOR-S6K pathway regulates REPS1 S709 phosphorylation in response to insulin stimulation. Instead, we observed that REPS1 phosphorylation is regulated via an MEK-ERK-RSK-REPS1 signaling axis. Our study validates REPS1 as a previously unrecognized signaling node downstream of the MAPK-RSK pathway mediating glucose uptake in skeletal muscle.

In our human cohort, we found a strong correlation between insulin-stimulated leg muscle glucose uptake and REPS1 S709 phosphorylation. Notably, 86% of the variance in leg glucose uptake could be explained by changes in REPS1 S709 phosphorylation. While this does not establish causality, our study provides evidence of impaired insulin-stimulated glucose uptake upon lowering REPS1 expression and S709 phosphorylation in muscle cells, strongly suggesting a direct role of REPS1 in insulin-stimulated muscle glucose uptake. In contrast, we did not find a correlation between REPS1 S709 phosphorylation and exercise-induced glucose uptake in mice or humans. This lack of association possibly reflects that, during exercise, other physiological factors—such as arterial delivery, interstitial availability, and intracellular metabolism of glucose— affect muscle glucose uptake, thereby reducing the relative importance of a single intracellular signaling event like REPS1 phosphorylation.^64^ Consistent with this, we demonstrated that the RSK-REPS1 axis is partially required for glucose uptake in response to contractions in glycolytic muscle. Since exercise activates multiple signaling pathways, its effects on glucose uptake are likely mediated by a variety of signaling events beyond REPS1 alone. This is further reflected in our dataset where exercise regulated approximately 50% of the identified phosphosites. An additional, unexplored role for REPS1 may lie in the insulin-sensitizing effects of exercise on GLUT4 trafficking, potentially through its involvement in endocytosis or vesicle sorting of GLUT4 into an insulin-responsive compartment.

Insulin-stimulated glucose uptake into peripheral tissues (e.g., muscle and adipose tissue) or inhibition of glucose production (liver) is compromised in the insulin-resistant state. Therefore, we hypothesized that insulin-stimulated phosphorylation of REPS1 at S709 would also be affected in these metabolically active tissues. Despite high expression of REPS1 in both the liver and white adipose tissue, insulin-stimulated phosphorylation of REPS1 at S709 was specific to skeletal muscle. This signaling was diminished in skeletal muscle from insulin-resistant mice. Given that skeletal muscle express high levelof GLUT4, and that the translocation of GLUT4-containing vesicles to the plasma membrane can be driven by multiple stimuli including muscle contractions,^65^ it is likely that skeletal muscle harbours a specialized signaling pathway governing GLUT4 trafficking..

Translating our findings in mice to human skeletal muscle, we observed considerable variability in insulin-stimulated REPS1 phosphorylation among individuals with NGT and those with T2D. Interestingly, this variability aligned closely with individual differences in overall insulin sensitivity. This alignment leads us to posit that REPS1 may serve as a pivotal signaling node in humans, holding substantial clinical relevance for metabolic diseases characterized by insulin resistance. In line with this, in published GWASs, we found that two independent lead variants (rs12193050 and rs2750415) were associated with the expression and pre-mRNA splicing in muscle and skeletal muscle tissue, respectively, implicating potential population heterogeneity in REPS1 (isoform) levels. Several genetic variants were related to lipid traits, lean mass, and T2D-related complications. While we anticipated more associations related to glycemic control, the limited methods for evaluating skeletal muscle insulin sensitivity might be a contributing factor. Comprehensive GWASs employing hyperinsulinemic clamp techniques are essential, as they could reveal previously undetected associations within genes like *REPS1*, providing a more detailed picture of muscle insulin sensitivity.

In conclusion, we mapped a comprehensive insulin- and exercise-induced phosphoproteome in skeletal muscle from healthy individuals. We identified a total of 233 phosphorylation events shared between the two stimuli with 71 residues on 55 proteins regulated in the same direction, many of which have a role in vesicle trafficking. We provide a comprehensive resource for future studies investigating the role of protein phosphorylation on skeletal muscle glucose uptake and metabolism. We highlight the conserved S709 residue of REPS1 as an insulin and exercise signaling convergence point in mouse and humans and demonstrate a tight association between this phosphorylation site and insulin-stimulated skeletal muscle glucose uptake. REPS1 binding partners and the putative upstream kinase, RSK, suggest a dual role in endocytosis and exocytosis regulation. The skeletal muscle-tissue-specific insulin response and impairment in insulin-resistant mice and humans emphasize the clinical relevance of REPS1 as a potential therapeutic target for diseases characterized with insulin resistance, such as T2D.

### Limitations of the study

The participants recruited for the phosphoproteome study were all young healthy men, who do not represent overall population heterogeneity including differences in gender, age, BMI, and ancestry. This initial focus allowed us to control for these factors and uncover valuable insights into insulin and exercise biology.

We provide initial evidence that REPS1 is phosphorylated in response to insulin and exercise; however, further studies are needed to elucidate the precise role of REPS1 phosphorylation in glucose uptake during and several time points into recovery of stimulation. Future work should also explore the involvement of REPS1 during recovery from exercise and how this may enhance insulin sensitivity. The exact mechanistic interplay between REPS1’s endocytotic and exocytotic functions remains to be fully understood. Investigating these pathways in more detail could provide additional insights into REPS1’s broader role in cellular regulation. While our findings emphasize the importance of the RSK-REPS1 axis, other regulatory inputs, including alternative kinases and phosphatases, may also influence REPS1 function and should be further explored. Lastly, although BI-D1870 is reported to be a highly specific RSK inhibitor, we cannot rule out the possibility that it may inhibit an unidentified kinase specifically expressed in skeletal muscle and involved in contraction-stimulated glucose uptake. Future studies generating an inducible muscle-specific REPS1 knockin mouse model is essential to complement the pharmacological data and provide deeper insights into the physiological role of REPS1 S709 phosphorylation in muscle-specific signaling and glucose uptake. Finally, the number of human participants included in the phosphoproteomic screen was limited, and the observed correlations between signaling markers such as REPS1 or AKT phosphorylation and leg glucose uptake should be interpreted with caution.

## Resource availability

### Lead contact

Requests for further information, resources, and reagents should be directed to and will be fulfilled by the lead contact Atul S. Deshmukh (atul.deshmukh@sund.ku.dk)

### Materials availability

This study did not generate new unique reagents.

### Data and code availability

• The MS proteomics data have been deposited at ProteomeXchange (http://proteomecentral.proteomexchange.org) via the PRIDE partner repository with the dataset identifier PXD046755 as of the date of publication.
• All original western blot and functional data are available in the Data S1 file.
• All original code has been deposited at https://github.com/fpm-cbmr/TINX_Phosphoproteomics_JKL and is publicly available at https://doi.org/10.5281/zenodo.15199640 as of the date of publication.
• Any additional information required to reanalyze the data reported in this paper is available from the lead contact upon request.

## Acknowledgments

Mass spectrometry analyses were performed by the Proteomics Research Infrastructure (PRI) at the University of Copenhagen (UCPH), supported by the Novo Nordisk Foundation (10.13039/501100009708NNF) (grant agreement number NNF19SA0059305). This work was supported by an unconditional donation from the 10.13039/501100009708Novo Nordisk Foundation (NNF) to NNF Center for Basic Metabolic Research (http://www.cbmr.ku.dk, 1 July 2018) (grant number NNF18CC0034900). The T2D study was supported by grants from the Region of Southern Denmark, the 10.13039/501100009708Novo Nordisk Foundation (grant number NNF15OC0015986), and the Sawmill Owner Jeppe Juhl and wife Ovita Juhl Memorial Foundation. M.G.-U. and T.O.K. were supported by the NNF grant number NNF22OC0074128. We want to thank Professor Amira Klip for kindly donating the L6-myc-GLUT4 cells used in the manuscript.

## Author contributions

Conceptualization, M.H., J.K., and A.S.D.; human clinical studies, J.V.S., K.H., M.H., J.B., K.E., S.J., A.K.L., and M.T.; investigation, J.K., C.B.L., M.G.-U., A.M.E., F.S., C.Y.Y., J.P.Q., J.H.S., L.S., M.T., A.G.-F., J.R.Z., T.O.K., and J.T.T.; writing – original draft, J.K. and A.S.D.; writing – review and editing, all; funding acquisition, A.S.D., M.H., and K.H.; resources, A.S.D. and M.H.; supervision, A.S.D. and M.H.

## Declaration of interests

The authors declare no competing interests.

## STAR★Methods

### Key resources table

**Table:** 

REAGENT or RESOURCE	SOURCE	IDENTIFIER
**Antibodies**		

REPS1	Cell Signaling Technology (CST)	Cat# 6404; RRID:AB_11220228
p-REPS S709	CST	Cat# 12005; RRID:AB_2797794
GAPDH	CST	Cat# 2118; RRID:AB_561053
*p*-AKT T308	CST	Cat# 9275; RRID:AB_329828
*p*-AKT S473	CST	Cat# 9271; RRID:AB_329825
AKT2	CST	Cat# 3063; RRID:AB_2225186
pan-AKT total	CST	Cat# 9272; RRID:AB_329827
*p*-ERK1/2 T202/Y204	CST	Cat# 9101; RRID:AB_331646
ERK1/2	CST	Cat# 4696; RRID:AB_390780
*p*-mTOR S2448	CST	Cat# 2971; RRID:AB_330970
mTOR	CST	Cat# 2972; RRID:AB_330978
*p*-AMPK T172	CST	Cat# 2531; RRID:AB_330330
AMPKα2	CST	Cat# 2532; RRID:AB_330331
ACC	CST	Cat# 3676; RRID:AB_2219397
p-P70S6K T389	CST	Cat# 9205; RRID:AB_330944
P70S6K	CST	Cat# 2708; RRID:AB_390722
*p*-RPS6 S235/236	CST	Cat# 2211; RRID:AB_331679
RPS6	CST	Cat# 2217; RRID:AB_331355
ACTIN	Sigma	Cat# A2066; RRID:AB_476693
FLAG	Sigma	Cat# F7425; RRID:AB_439687
*p*-ACC S221	Millipore	Cat# 07-303; RRID:AB_310504
GLUT4	Thermo Scientific	Cat# PA1-1065; RRID:AB_2191454

**Bacterial and virus strains**		

Mouse Flag-*Reps1/Control Aav1*	VectorBuilder	NM_009048.2

**Biological samples**		

Human skeletal muscle biopsies	This paper	N/A
Mouse skeletal muscle, liver and adipose tissue	This paper	N/A

**Chemicals, peptides, and recombinant proteins**		

^3^H-2-Deoxyglucose	PerkinElmer	Cat# NET549A005MC
Ultima Gold scintillation liquid	PerkinElmer	Cat# 6013329
2-Chloroacetamide (CAA)	Sigma	Cat# 22790
Tris base	Sigma	Cat# T6066
NaCl	Sigma	Cat# 31434
Tween 20	Sigma	Cat# P7949
Glycine	Sigma	Cat# G7126
Trifluoroacetic acid	Sigma	Cat# 808260
KH2PO4	Sigma	Cat# P5655-100G
Acetic acid	Sigma	Cat# 71251
ECL luminata forte western HRP substrate	Merck	Cat# WBLUF0500
Bovine serum albumin	Merck	Cat# A7906
Skim milk powder	Merck	Cat# 70166
NH4OH	Merck	Cat# 105428
Dithiotreitol (DTT)	Merck	Cat# 43815
Sodium dodecyl sulfate (SDS) solution	Merck	Cat# 71736
4–20% Criterion™ TGX™ Precast Midi Protein Gel, 26 well, 15 μL	BioRad	Cat# 5671095
4–20% Criterion™ TGX™ Precast Midi Protein Gel, 18 well, 30 μL	BioRad	Cat# 5671094
Trans-Blot Turbo Midi 0.2 μm PVDF Transfer Packs	BioRad	Cat# 1704157
2-Mercaptoethanol	BioRad	Cat# 1610710
10x Tris/Glycine/SDS	BioRad	Cat# 1610772
4x Laemmli Sample Buffer	BioRad	Cat# 1610747
Acetonitrile	Thermo Scientific	Cat# A9554
TCEP, neutral pH	Thermo Scientific	Cat# 77720
Tandem-mass tag (TMT)	Thermo Scientific	Cat# A34808
BI-D1870	MedChemExpress	Cat# HY-10510
MP-7	MedChemExpress	Cat# HY-14440
PF-4708671	MedChemExpress	Cat# HY-15773
U0126	CST	Cat# 9903
Rapamycin	Selleckchem	Cat# S1039

**Deposited data**		

Phosphoproteomics data of human skeletal muscle	This paper	PRIDE: PXD046755
Data S1	This paper	Raw data underlying graphs
PhosphoSitePlus® Regulatory sites Database	https://www.phosphosite.org/	N/A
R code	This paper	https://doi.org/10.5281/zenodo.15199640

**Experimental models: Cell lines**		

C2C12 myoblasts		N/A
L6 rat myoblasts	Lab of Professor Amira Klip	N/A

**Experimental models: Organisms/strains**		

Mouse C57BL/6NTac	Taconic	N/A

**Oligonucleotides**		

ON-TARGETplus siRNA Non-targeting Control Pool	Horizon	Cat# D-001810-10-05
ON-TARGETplus siRNA SMARTPool mouse Reps1	Horizon	Cat# L-040836-01-0005

**Software and algorithms**		

R version 4.2.2	R Development Core Team	https://www.R-project.org/
Maxquant v2.0.1.0	Cox et al.^66^	https://www.biochem.mpg.de/5111795/maxquant
limma v3.54.2	Ritchie et al.^67^	https://doi.org/10.18129/B9.bioc.limma
Kinase enrichment analysis	Johnson et al.^34^	https://kinase-library.phosphosite.org/
PyMOL	Schrödinger	https://pymol.org/
Image Lab	Bio-Rad Laboratories	Version 4.0
GraphPad Prism	GraphPad Software	https://www.graphpad.com/

### Experimental model and study participant details

#### Subjects and eligibility criteria

Eight healthy male volunteers took part in the study and were selected from a sub-group of a larger study.^68^ Prior to inclusion, subjects met for an assessment of eligibility criteria. The criteria were: healthy males, 18–36 years of age, non-smoker, engaged in physical activity for 2–5 h/week, exhibit maximum oxygen uptake (40–60 mL/kg/min, lean mass 55–65 kg or lean mass index 14–22 kg/m^2^, no usage of beta2-agonists or other prescription medicine, and no allergy toward study medication. Withdrawal criteria included experiencing unacceptable side effects, complications related to the study, or non-compliance with the protocol. The study adhered to the 2013 Helsinki Declaration and was approved by the ethics committee of Copenhagen, Denmark (H-4-2014-002). All subjects provided oral and written informed consent before being included in the study. Body composition was assessed by dual-energy X-ray absorptiometry (Lunar DPX IQ, Version 4.7 E, Lunar Corporation, Madison, WI, USA) (DXA) followed by incremental cycling to exhaustion on a bike ergometer for assessment of VO2max by indirect calorimetry (Monark LC4, Monark Exercise AB, Vansbro, Sweden) as previously described.^69^

For the T2D study, a subset of 10 patients with type 2 diabetes from the “specialist supervised individualized multifactorial treatment of clinically diagnosed type 2 diabetes in general practice (IDA)” study^70^ and 10 persons with normal glucose-tolerance matched on gender, BMI, age and smoking took part in the T2D study. All participants had normal results on blood screening tests and ECG. Informed consent was obtained from all subjects before participation. The study was approved by the Regional Scientific Ethical Committees for Southern Denmark (Projekt-ID: S-20120186) and was performed in accordance with the Helsinki Declaration.

#### Animal model

All animal experiments were approved by the Danish Animal Experiments Inspectorate (License no. 2017-15-0201-01276 & 2018-15-0201-01493). All mice were male C57BL/6NTac, aged 7–16 weeks, and housed under a standard 12:12-h light–dark cycle.

### Method details

#### Hyperinsulinemic euglycemic clamp trial

Subjects arrived at the laboratory in a fasting state for assessment of insulin-stimulated whole-body glucose disposal during a hyperinsulinemic-euglycemic clamp. They refrained from vigorous physical activity and alcohol for 48 h and caffeine and nicotine for 24 h. Before the clamp, subjects ingested four to five potassium tablets to prevent hypokalaemia (Kaleorid, 750 mg KCl, Karo Pharma, Sweden) and had a catheter placed in the antecubital vein for infusion of glucose and insulin. In addition, catheters were inserted in the femoral vein and artery and a muscle biopsy was sampled during local anesthesia. The clamp was initiated with a 1 min priming dose of 53.5 pmol/kgbw insulin (100 IU/mL, Novo Nordisk, Copenhagen, Denmark) and continued for 120 min with a constant insulin infusion of 8 pmol/kgbw/min. Before the clamp and every 5 min during the clamp, femoral venous and artery blood samples were collected for assessment of glucose concentration and femoral artery blood flow was measured for assessment of leg glucose balance.

Furthermore, blood samples were collected before and during the clamp for determination of plasma insulin. Glucose was infused during the clamp from a 20% glucose solution (Fresenius Kabi, 200 mg/mL) to maintain euglycaemia at ∼5 mM. At the end of the clamp, another muscle biopsy was sampled.

For the T2D study, an HEC was performed after an overnight fast. The participants were instructed to refrain from physical activity 48 h prior to test. In patients with T2D, glucose, lipid, and blood pressure lowering medication was withdrawn one week prior to the clamp studies. In brief, the clamp consisted of a 2-h basal period with infusion of a primed constant amount of [3-3H]-tritiated glucose to obtain tracer equilibration. This was followed by a 4-h insulin-stimulated period using an insulin infusion rate of (40 mU · m-2 · min-1) to achieve euglycemia (5.0–5.5 mmol/L) in the insulin-stimulated steady-state period. Skeletal muscle biopsies were obtained before and after the insulin infusion period under local anesthesia (lidocaine).^65^

#### Cycle exercise trial

On a separate day in a fasting state, a resting muscle biopsy was obtained under local anesthesia (2 mL lidocaine without noradrenaline (epinephrine), Xylocaine 20 mg/mL, AstraZeneca, Cambridge, UK). Hereafter, subjects completed a 10-min bike ergometer exercise with the highest possible effort. Immediately after the exercise, another biopsy was sampled. Hyperinsulinemic-euglycemic clamp trial and the exercise trials (in the same eight individuals) were separated by wash out period of around 1–2 weeks.

#### Blood analyses

Blood samples were drawn in EDTA tubes and stored on ice for 30 min before they were spun at 4°C and 3000 rpm for 5 min, after which plasma was collected and stored at 80°C until analysis for catecholamines, insulin, and FFAs. Plasma adrenaline and noradrenaline were determined with a commercially available ELISA kit (2-CAT plasma ELISA high sensitivity kit; Labor Diagnostica Nord, Nordhorn, Denmark) according to the manufacturer’s instructions. Plasma insulin was determined with ELISA (Dako Denmark) according to manufacturer’s instructions. Plasma FFA was determined with ELISA (NEFA-HR, R1 and R2 sets; TriChem ApS–Interkemi, Skanderborg, Denmark) according to the manufacturer’s instructions.

#### One-legged knee extensor exercise

Participants met in the morning after an overnight fast. Upon arrival at the laboratory, subjects rested in a supine position and catheters were inserted into the femoral artery and vein during local anesthesia (20 mg/mL lidocaine without adrenaline, Xylocaine; AstraZeneca, London, UK). Incisions were made through the skin and fascia at the belly of the vastus lateralis muscle for sampling of biopsies. After 30 min of rest in a sitting position, participants were moved to a knee extensor ergometer,^71^ where their right foot was strapped to a boot that was connected to the ergometer crank. Participants then performed a brief warmup, followed by a bout of exercise at 90% of maximal incremental peak power output until exhaustion. During exercise, participants were instructed to contract during the concentric phase of movement while relaxing during the eccentric phase to minimize involvement of hamstring muscles. Participants maintained a cadence of 60 RPM and exhaustion was defined as inability to maintain 60 RPM for >5 s during strong verbal encouragement.

Femoral arterial and venous blood samples were drawn before exercise, immediately following exhaustion, and 1–10 min in recovery. Samples were drawn in heparinised tubes for immediate analyses of glucose using an ABL800 Flex (Radiometer, Copenhagen, Denmark). Femoral artery blood flow was measured at the same time as blood sample collection.

A muscle biopsy was sampled from the vastus lateralis using the Bergström needle procedure^72^ before and immediately following exercise.

#### Blood flow measurements

Blood flow was measured by ultrasound Doppler (Logic E9, GE Healthcare, Illinois, US) equipped with a linear probe operating with imaging frequency of 9 MHz and Doppler frequency of 4.2–5.0 MHz. The site of blood velocity measurements was at the common femoral artery below the inguinal ligament but above the bifurcation to the superficial and profound branches to avoid turbulence from the bifurcation. Doppler traces were recorded and averaged over 10 s with the midpoint of the sampling period being the time of blood sampling.

#### Calculation of leg glucose uptake

Leg glucose uptake was calculated from femoral arterial blood flow and the arteriovenous difference in glucose plasma concentrations:

$$glucose\;uptake=BF\times \left({glucose}_{a}-{glucose}_{v}\right)$$

where *BF* is blood flow in L/min, *glucose*_*a*_ and *glucose*_*v*_ are arterial and venous, respectively, plasma glucose concentrations in mmol/L.

#### Muscle biopsies

All skeletal muscle biopsies were sampled using the suction-modified Bergström needle technique.^72^ This technique is widely used because it is safe, minimally invasive, and provides adequate material for biochemical analyses (typically 100–150 mg wet wt).^73^ Before sampling, we applied local anesthesia (2–3 mL lidocaine without epinephrine, Xylocaine, 20 mg/mL, AstraZeneca, Cambridge, UK) through the skin at the belly of the vastus lateralis muscle. Next, we made a small incision (3 mm) and nicked the muscle fascia using a sterile surgical blade. Through this incision, we collected the biopsy using a 4-mm Bergström needle (Stille, Stockholm, Sweden) with suction. Immediately after sampling, we washed the biopsy in sterile saline solution (0.9% NaCl, Fresenius Kabi, Sweden) and quickly removed visible blood, connective tissue, and fat after which we froze the sample in liquid nitrogen and stored it at −80°C until analysis.

#### *In vivo* mouse insulin stimulation

7-week old C57BL/6NTac male mice were fed *ad libitum* an LFD (Research diet:D12450Ji) or HFD diet (Research Diet:D12451i) for 16 weeks on a normal 12:12-h light dark cycle. On the day of termination, fed mice were anesthetized by an intra-peritoneal injection of pentobarbital (10mg/100mL) and injected retro-orbitally with either saline or human insulin (1U/kg). After 15 min, tissues were immediately removed and frozen in liquid nitrogen until further analysis. Blood glucose levels were measured pre/post saline/insulin injection by a glucometer.

#### *In situ* mouse contractions

Mice (male, C57BL/6NTac; 12–16 weeks of age) were anesthetized with pentobarbital (2.5 mg/mouse) and kept warm on a heating mat throughout the experiment. The sciatic nerve was exposed and stimulated (0.5 s train every 1.5 s at 5V, 0.1ms, 100hz) to contract the lower leg muscles. The contralateral leg to the contracted leg was sham operated and serves as a non-contracted control. A retro-orbital injection was administered immediately prior to nerve stimulation (15 mU insulin or saline, 3 mg glucose, 20 μCi [3H]2-deoxyglucose in gelofusine) and tissues were dissected 15 min after glucose tracer, insulin/saline injection and the initiation of contraction. The TA muscle was quickly dissected and snap frozen. Muscles were processed as in.^74^ Protein concentration was determined by the BCA-assay (Thermo #23225). Muscle protein lysate was mix with either 0.3M Ba(OH)_2_ and ZnSO_4_ or 4.5% HClO_4_ before centrifugation at 10000 RPM for 5 min at room temperature. Supernatant was collected and added to scintillation vials with Ultima Gold scintillation fluid (PerkinElmer #6013329) before counted on a Hidex 300 SL scintillation counter. Blood was collected every 5 min and specific activity calculated as in.^74^

#### Ex-vivo mouse muscle insulin-stimulated glucose uptake

Soleus muscle from male mice (C57BL/6NTac; 12–16 weeks of age) was rapidly dissected, as previously described,^75^^,^^76^ and incubated into warmed (30°C) and oxygenated (95% O_2_, 5% CO_2_) Krebs-Ringer buffer (KRB, 117 mM NaCl, 4.7 mM KCl, 2.5 mM CaCl2, 1.2 mM KH2PO4, 1.2 mM MgSO4, 24.6 mM NaHCO3) with 0.1% bovine serum albumin (BSA), and 5 mM glucose in a myograph system (820MS DT, Denmark). Muscles were pre-incubated for 60 min in KRB containing BSA, 8mM mannitol, and 2mM pyruvate 10uM BI-D1870 or vehicle (DMSO). The media was then changed for a 30 min incubation with insulin (300μIU/ml, Actrapid, Novo Nordisk, Denmark). After 20 min, the buffer was changed to a buffer containing 1 mM of 2-DG and 3H-2-DG (0.0185 MBq/mL, PerkinElmer, #NET549A005MC), 7 mM of mannitol and 14C-mannitol (0.0167 MBq/mL, PerkinElmer, #NEC314250UC) for 10 min. After which, muscles were harvested and snap frozen in liquid nitrogen. Muscles were processed as above and supernatant protein lysate counted on a Hidex 300 SL scintillation counter. All muscle counts were related to the specific activity in each myograph chamber.

#### Ex-vivo muscle contraction-stimulated glucose uptake

EDL muscle from male mice (C57BL/6NTac; 12–16 weeks of age) was isolated, incubated, and processed as described above. Optimal muscle length was determined by finding the length of maximal isometric twitch response (pulse voltage 14V, pulse width 0.5ms). Ten-minute muscle fatiguing contraction protocol was carried out with a pulse voltage of 14V, pulse width of 0.2ms, interval of 6.50ms, frequency of 150hz, 75 pulse counts in each train, 500ms total train time, and 5,000ms pause between trains for a total of 110 trains. Glucose uptake was measured as described above immediately after contractions for 10 min.

#### Cell culture

C2C12 cells were cultured in Dulbecco's Modified Eagle Medium (DMEM #41965-039) supplemented with 10% Fetal Bovine Serum and 1% penicillin streptomycin (P/S). Cells were maintained in an incubator with 37°C and 5% CO_2_. C2C12 myoblasts were grown to ∼80% confluence before initiating differentiation with DMEM supplemented with 2% Horse serum and 1% P/S. All cell lines were tested negative for mycoplasma contamination.

#### Cell signaling experiments

C2C12 myotubes were serum-starved for 4 h before pre-incubating cells with inhibitors (10μM BI-D1870, 10μM U0126, 100nM Rapamycin, 10μM PF-4708671 and 10μM MP-7) for 20 min followed by 10 min stimulation of 100nM insulin. Cells were then washed twice in ice-cold phosphate buffered saline (PBS) and lysed in 2% SDS, 50mM Tris pH = 7.4. Lysate was then boiled for 5 min at 95°C. A small aliquot was used for DC assay protein determination. Sample concentration was adjusted with SDS-buffer and 4x sample buffer (10% 2-mercaptoethanol, 13.3% glycerol and 444mM Tris-HCl pH = 6.8).

#### SiRNA-mediated knockdown

On the same day as differentiation was initiated, cells were transfected with siRNA against *Reps1* (Dharmacon #L-040836-01-0005) or scramble siRNA (Dharmacon #D-001810-10-20). Complexes of Transit-X2 (Mirus #MIR6000), Opti-MEM and siRNA were prepared in a concentration of 25nM and incubated at room temperature for 20 min before the siRNA complexes were added to the cells. The culture media was changed every ∼48 h. Knockdown of *Reps1* was later confirmed by Western Blot.

#### Aav transduction

Mouse *Reps1* (NM_009048.2, VectorBuilder) with N-terminal FLAG tag was subcloned into Aav serotype 1 vector under a CMV promoter. The control-FLAG contained a random open reading frame stuffer similarly under a CMV promoter. Vectors were added at a concentration of 10x10ˆ5 per well in a 6-well plate at day of differentiation induction. The culture media was changed every ∼48 h.

#### *In vitro* glucose uptake assay

Glucose uptake of C2C12 myotubes was measured with a ^3^H-2-Deoxyglucose (2DG) assay on day 6 of differentiation. Cells were serum starved for 4 h in serum free DMEM with 1% P/S prior to the assay. After serum starvation cells were washed one time in pre-warmed Krebs Ringer HEPES buffer (140 mM NaCl, 4.7 mM KCl, 2.5 mM, 1.25 mM, 1.2 mM HEPES, 0.2% bovine serum albumin (BSA), pH = 7.4) before incubation with KRHepes or 100 nM insulin for 30 min. Thereafter, cells were incubated for 5 min with ^3^H-2DG (0.4 μCi/mL) and 50μM 2DG. Glucose uptake was stopped by washing cells with ice-cold KRHepes buffer while the plate was on ice. Cells were then lysed in 2% SDS and boiled for 5 min at 95°C. 100 μL cell lysate was added to 3 mL of Ultima Gold scintillation liquid (PerkinElmer #6013329). Samples were kept in the dark overnight and ^3^H-2DG uptake was measured the following day on a Hidex 300 SL scintillation counter. ^3^H-2DG uptake was subsequently adjusted for protein content using a detergent compatible (DC)-assay (Bio-Rad). The glucose-uptake experiment was repeated on three separate days with same observed effect. For the RSK-inhibitor glucose uptake experiment, cells were preincubated with 10 μM BI-D1870 for 20 min before 10 min of 100nM insulin stimulation.

#### GLUT4-translocation assay

L6 muscle cells stably transduced with myc-GLUT4 were grown in αMEM medium containing 1% P/S, 10% FBS, and 2μm/mL blasticidin until they reached 70% confluency. The cells were then seeded into a 96-well plate (10,000 cells/well), and differentiation was initiated on the day of confluency in medium with 1% P/S and 2% FBS. On the day of the experiment (day 6), differentiated myotubes were serum-starved for 2 h. Cells were pre-incubated with either DMSO or 10 μM BI-D1870 for 20 min, followed by stimulation with 100 nM insulin for 10 min. After treatment, the cells were immediately placed on ice and washed twice with ice-cold PBS before being fixed with 3% paraformaldehyde for 10 min. Fixation was quenched using 0.1 mM glycine for 5 min, followed by three washes with PBS+ (PBS containing 0.9 mM Ca^2+^ and 0.49 mM Mg^2+^). The cells were blocked with 5% milk for 10 min and then incubated with the primary anti-myc antibody (Sigma-Aldrich #C3956, 1:1000 in 5% milk) for 60 min at room temperature. After five washes with PBS+, the cells were incubated with HRP-conjugated goat anti-rabbit antibody (1:1000 in 5% milk, Bio-Rad: #1706515) for 30 min, followed by five additional washes with PBS+. One OPD tablet (Sigma-Aldrich #5412) was dissolved in 0.05 M phosphate/citrate buffer (pH = 5), supplemented with H_2_O_2_, and added to the cells for 30 min in the dark. Absorbance was measured spectrophotometrically at 450 nm using a Hidex plate reader. Cells incubated without the primary antibody were used as controls to subtract background noise. The GLUT4-translocation experiment was repeated on three separate days, consistently showing the same effect.

#### Sample processing

##### Phosphoproteomics

Freeze-dried muscle was powdered and lysed in 4% sodium dodecyl sulfate (SDS), 100mM Tris pH = 8.5 with an Ultra Turrax homogenizer (IKA). Samples were immediately boiled for 5 min, tip-probe sonicated and spun down at 16,000g for 10 min. The resultant supernatant was protein precipitated using four times the volume of −20°C acetone overnight. The protein pellets were washed 3 times in acetone, air-dried and resolubilized in 4% sodium deoxycholate (SDC), 100mM Tris pH = 8.5 with a bioruptor sonicator (30s on/off, 15 cycles). Protein concentration was determined using Bicinchoninic acid (BCA) assay and 1mg of protein was subsequently reduced and alkylated for 5 min at 40°C with 5mM tris-(2-carboxyethyl)phosphine (TCEP) and 40mM 2-chloroacetamide (CAA), respectively. Enzymatic digestion was initiated by adding Trypsin and lysC at a 1:100 enzyme:protein ratio and the mixture was digested overnight at 37°C. Protein digestion was stopped by adding 1% trifluoroacetic acid (TFA) and precipitate was cleared by centrifugation at 20,000g for 10 min. Supernatant was desalted on C18 cartridges (Sep-Pak, waters) and eluted with 50% acetonitrile (ACN). Peptides were then vacuum dried and concentration was determined by measuring the absorbance at 280/260 (NanoDrop, Thermo Scientific). 200μg of peptides in 100mM HEPES pH = 8-5 were then labeled with 400μg of 11-plex TMT (Thermo, #A34808) for 1 h followed by 15 min of quenching with 0.27% NH2OH. Peptides pre/post exercise (study day 1) or insulin (study day 2) were labeled on two separate 11-TMT-plexes. A pool of peptides across conditions were placed in the last channel 131C. An aliquot of each sample was tested for near-complete labeling efficiency (>99% labeling).

TMT-peptide pools were then desalted on C18 cartridges and eluted with 50% ACN. Prior to phospho-enrichment, the samples were adjusted to 6% TFA, 3mM KH2PO4, and 50% ACN. TiO_2_-beads (GLsciences) were equilibrated in 80% ACN and 6% TFA. Equilibrated TiO_2_-beads were added in a ratio of 12:1 (bead to peptide) for 15 min rotating end-over-end at room temperature. Supernatant was passed through a second and third round of enrichment in an 8:1 and 4:1 bead to peptide ratio, respectively. Beads were combined and washed four times in 60% ACN and 1%TFA. Beads were then transferred in 80% ACN and 0.5% acetic acid on top of 2xC8 layer plugged into a p200 tip. Captured phosphopeptides were then eluted in 80% ACN, 1.25% NH4OH and vacuum dried for 20 min. 1% TFA was added to the samples and loaded on to equilibrated three layered Styrene-DivinylBenzene – Reversed Phase Sulfonate (SDB-RPS) StageTips. The phosphopeptides were subsequently washed in 1% TFA in isopropanol followed by two washes with 0.2% TFA. Lastly, phosphopeptides were eluted in 80% ACN and 1.25% NH4OH, vacuum dried and resuspended in 20uL 5% ACN and 0.1% TFA.

#### Immunoprecipitation and interactome analysis

Upon C2C12 myoblast differentiation, cells were transduced with 100,000 virus per well for 48 h before media change. On day of harvesting, cells were starved for 3 h and lysed in ice-cold lysis buffer (1% Triton X-100, 150mM NaCl, 50mM Tris pH = 7.4, 1mM EDTA, 6mM EGTA) supplemented with protease and phosphatase inhibitors (Roche). Cell homogenate was incubated end-over-end for 45 min before centrifugation at 16,000G for 10 min at 4°C. Protein concentration was determined by the BCA assay. A total of 200μg of protein lysate per sample was used for either FLAG- or endogenous IP. For IP, appropriate antibody was added to lysate and left overnight. The following day protein G agarose beads were added and samples incubated for 2 h. Beads were washed two times in lysis buffer followed by two washes in 150mM NaCl, 50mM Tris pH = 7.4. Proteins were then eluted, reduced and alkylated from beads in 2M Urea, 1mM dithiothreitol (DTT) and 0.5μg trypsin for 1 h. Supernatant was collected and alkylated with 5mM 2-Iodoacetamide (IAA) and digested overnight at 37°C. The following day, digestion was stopped by adding 1% TFA and 20% of eluate was loaded onto equilibrated Evotips ready for measurement.

#### Liquid chromatography mass spectrometry (LC-MS/MS)

##### Phosphoproteome analysis

Desalted and TMT11plex-labeled peptides were resuspended in buffer A∗ (2% ACN, 0.1% TFA), and fractionated into 16 fractions by high-pH fractionation. In short, 4μg of peptides were loaded onto a 30 cm × 0.25mm Pepsep 1.9um ReproSil C18 120 Å column via an EASY-nLC 1200 HPLC (Thermo Fisher Scientific) in Spider buffer A (1% tetraethylammonium fluoride (TEAF), 0.1% tetraethylammonium (TEA)). Peptide separation was done with a non-linear gradient of 5–44% Spider buffer B (1% TEAF, 0.1% TEA, 80% ACN) at a flow rate of 1.5 μL/min over 62 min. Collection of fractions was done at 60 s interval with a concatenation workflow to achieve a total of 16 fractions. All fractions were then evaporated, and peptides resuspended in buffer A∗, and measured using an EASY-nLC 1200 HPLC system (Thermo Fisher Scientific) coupled through a nano-electrospray source to a Tribrid Eclipse mass spectrometer (Thermo Fisher Scientific). Peptides were loaded in buffer A (0.1% formic acid (FA)) and separated on a 25 cm column Aurora Gen1 (kept at 50°C), 1.6uM C18 stationary phase (IonOpticks) with a non-linear gradient of 5–44% buffer B (0.1% FA, 80% ACN) at a flow rate of 400 nL/min over 91 min with a total gradient time of 101 min including washing. Spray voltage was set to 2400 V. Data acquisition switched between a full scan (120K resolution, 50ms max. injection time, AGC target 100%) and 3 s cycle time-controlled data-dependent MS/MS scans (50K resolution, 120ms max. injection time, AGC target 200% and HCD activation type). Isolation window and normalized collision energy were set to 0.7 and 35, respectively. Precursors were filtered by charge state of 2–6 and multiple sequencing of peptides was minimized by excluding the selected peptide candidates for 45 s. The PrecursorFit filter, Fit error 70% in a window of 1.2Da, was also activated.

#### Interactome analysis

For the interactome experiment, the peptides were subjected to separation using a 15 cm column with an internal diameter of 150 μM, which was packed with 1.9 μm C18 beads (Pepsep brand). This separation process was carried out on an Evosep ONE HPLC system, employing the specific protocol designed for '30 samples per day', and peptides were introduced into the system via a CaptiveSpray source equipped with a 20 μm emitter. The mass spectrometric analysis was performed using a timsTOF SCP mass spectrometer from Bruker, functioning in the parallel accumulation-serial fragmentation (PASEF) mode.^77^ To elaborate, the DDA-PASEF scan for both MS and MS/MS was configured to encompass a range from 100 to 1700 m/z. The TIMS mobility spectrum was set within the bounds of 0.6–1.6 (V cm−2). Both TIMS ramp and accumulation times were precisely fixed at 100 ms. During each cycle, which lasted a total of 1.17 s, 10 PASEF ramps were recorded. The MS/MS target intensity was defined at 20,000, with an intensity threshold at 1,000. Furthermore, a stringent exclusion list was applied, set at 0.4 min for precursors within a narrow window of 0.015 m/z and 0.015 V cm−2.

#### Data analysis

##### Raw file processing

Raw files were quantified in MaxQuant v2.0.1.0 against the reviewed human fasta (09082021).^66^ Default settings were applied including a minimum peptide length of seven amino acids. Deamidation (NQ), methionine-oxidation (M) and N-terminal acetylation were set as fixed modifications. Phospho (STY) was set as a variable modification. For interactome analysis, raw files were processed in Fragpipe (v.20.0) with default settings and MaxLFQ enabled.^78^

#### Bioinformatics analysis

Maxquant output STY-file was imported into Perseus (2.0.7.0)^79^ and phosphopeptides expanded to represent phosphosites. Data was then imported into R studio v4.2.1 and log2-transformed. Data were median-scaled and unwanted variation was removed by normalizing to stably phosphorylated sites with the phosR package.^80^ The data were filtered to have no missing values. To identify differentially regulated phosphosites, we applied limma with ebayes data smoothening.^67^ The linear model was blocked for subjects. *p* values were adjusted with the Benjamini Hochberg (BH) method and adjusted *p* values <0.05 were considered statistically significant.

Kinase-activation analysis for Ser/Thr kinase was performed by the online tool: https://kinase-library.phosphosite.org/site.^34^ Sequence windows were uploaded with phosphorylated residue centralized. Kinases were then filtered to be expressed in human skeletal muscle as part of the human protein atlas database (∼13000 transcripts).^35^ Regulatory- and disease-associated sites were downloaded from PhosphositesPlus.^81^ For each subcategory a two-sided fishers exact test was applied.

#### Go enrichment analysis

Gene-ontology enrichment analysis was done with the clusterProfiler package.^82^ Enrichment was done on the gene-level. Whole phosphoproteome and interactome data was used as background. BH adjusted *p* values <0.05 were considered significant.

#### Alphafold-structure

Predicted 3-dimensional structure of CLASP2 was extracted from alphafold and specific phosphorylated residues were edited in the Pymol software.^83^

#### Interactome data analysis

The combined_protein output file was imported into R studio. Data were log2-transformed and filtered for quantification in at least 50% (3 samples) within a group. Missing values were imputed from a normal distribution with a mean downshift of 1.8 and standard deviation of 0.3. A two-sided t-test was used to assess differences between groups. BH adjusted *p* values <0.05 were considered significant.

#### Querying genetic associations of REPS1 variants

To identify which traits are associated with the *REPS1* gene, we searched for associations between *REPS1* variants and complex traits and diseases in genome-wide association studies (GWASs) by querying “*REPS1*” in the NHGRI-EBI GWAS catalog^55^ on October 2, 2023. To assess whether the reported associations belong to independent signals, we computed a linkage disequilibrium (LD) matrix using the LDLinkR package (v1.4.2)^56^^,^^57^. We considered that variants represent independent signals if the r^2^ between the variants was <0.1 within ±500kb. For each independent signal, we identified the lead variant with the lowest *p* value and queried it in Open Target Genetics^59^ to obtain additional associations from a different source (*P* < 1x10^−6^). We also assessed whether these variants were significantly associated with the mRNA expression (eQTL) or splicing (sQTL) of *REPS1* across different tissues by querying Open Target Genetics using otargen R package (v.1.0.0).^84^

#### Immunoblot analysis

##### Western blotting of animal tissues

Tissues were powdered and lysed in 4% SDS, 100mM Tris pH = 7.4 with an Ultra Turrax homogenizer (IKA) and immediately boiled. Samples were tip-probe sonicated and centrifuged at 16,000g for 10 min. Supernatant was collected and protein concentration determined by the DC-assay. 4x Sample-buffer (10% 2-mercaptoethanol, 13.3% glycerol and 444mM Tris-HCl pH = 6.8) was added to lysate and boiled again for 5 min at 95°C.

#### Western blotting of human samples

Powdered muscle was lysed in ice-cold homogenization buffer (10% glycerol, 20 mM Na-pyrophosphate, 150 mM NaCl, 50 mM HEPES (pH 7.5), 1% Igepal, 20 mM β-glycerophosphate, 2 mM Na3VO4, 10 mM NaF, 2 mM phenylmethylsulfonyl fluoride (PMSF), 1 mM ethylenediaminetetraacetic acid (EDTA) (pH 8), 1 mM ethylene glycol-bis(β-aminoethyl)-N,N,N,N-tetraacetic acid (EGTA) (pH 8), 10 μg⋅ml–1 Aprotinin, 10 μg⋅ml–1 Leupeptin and 3 mM Benzamidine) with steel beads at 28.5 Hz for 1 min (Qiagen TissueLyser II, Retsch GmbH, Haan, Germany). Homogenate was then incubated for 1 h end-over-end at 4°C followed by centrifugation for 20 min at 18,320g and 4°C. Protein concentration was determined by the BCA assay.

15μg was loaded on a 4–20% acrylamide gradient gel with 18 or 26-wells (Bio-Rad #5671094 & Bio-Rad #5671095) and separated for 20 min at 100v followed by 150V for 1 h. Proteins were transferred with a semi-dry transfer system (Bio-Rad) onto a polyvinedylene difluoride (PVDF)-equilibrated membrane (Bio-Rad turbo-transfer #1704157) for 7 min at 25V. Membranes were blocked in either 2% milk or 3% BSA in Tris-buffered saline (TBS, pH7.4) supplemented with 0.05% tween 20 for 1 h and incubated overnight with primary antibody on a rocking platform. The following day, membranes were washed 3 times in TBS-T and incubated for 45 min with secondary antibody (Bio-Rad: Goat Anti-rabbit #1706515 or Goat-anti mouse #1706516). Lastly, membranes were washed 3x every 10 min for a total of 3 times before ECL (Millipore #WBLUF0500) was added and imaged by chemidoc+ system (Bio-Rad). Immunoblots were analyzed in ImageLab software.

#### Antibodies

REPS1 (CST #6404), p-REPS S709 (CST #12005), GAPDH (CST #2118), *p*-AKT T308 (CST #9275), *p*-AKT S473 (CST #9271), AKT2 (CST #3063), pan-AKT total (CST #9272), *p*-ERK1/2 T202/Y204 (CST #9101), ERK1/2 (CST #4696), mTOR (CST #2972), *p*-mTOR S2448 (CST #2971), *p*-AMPK T172(CST #2531), AMPKα2 (CST #2532), ACC (CST #3676), p-P70S6K T389 (CST# 9205), P70S6K (CST #2708), *p*-RPS6 S235/236 (CST #2211), RPS6 (CST #2217), ACTIN (sigma A2066), *p*-ACC S221 (Millipore 07–303), GLUT4 (Thermo PA1-1065), FLAG (sigma, F7425).

### Quantification and statistical analysis

For analysis of paired human samples a two-sided paired samples t-test was used. For two-factor designs a two-way ANOVA analysis was used with or without repeated measures. Tukeys or Sidak multiple comparison test were used as post hoc test dependent on repeated measures. For unpaired two condition design a two-sided two sample t-test was used. Pearson’s correlation was used to analyze association between two independent variables (normality tested with the Shapiro-Wilk test *p* > 0.05). In Figure 5F, an ANCOVA model was used to predict GIR (dependent variable) by Group (NGT/T2D) and delta (post-pre clamp) p-REPS1 levels (independent variables). Additionally, the model included an interaction term to examine the potential differential effect of p-REPS1 levels on GIR across the two groups (NGT/T2D). All analyses were performed in R Studio or Prism software. *p* values <0.05 were considered significant.

## Supplemental information

Document S1. Figures S1–S5

Table S1. Data related to the analysis of human skeletal muscle phosphoproteomics (A) Phosphosite level information in basal and insulin-stimulated states. (B) Phosphosite level information at rest and immediately post-exercise. (C) Phsophosites overlapping between insulin and exercise data matrices. (D) Phosphosites significantly regulated in response to insulin and exercise. (E) Predicted activated kinases during insulin stimulation in human skeletal muscle. (F) Predicted activated kinases during exercise in human skeletal muscle

Table S2. Genetic associations with REPS1 expression and regulation (A) Results of REPS1 query in GWAS Catalog. (B) Associations with the lead SNPs reported in (A) Open Targets Genetics. (C) Results of querying the 17 REPS1 variants in Open Targets Genetics for eQTL and sQTL REPS1 associations

Data S1. Unprocessed source data underlying all graphs, related to Figures 1–6 and S1–S5

Document S2. Article plus supplemental information
