# Supplementary material for: Insulin- and exercise-induced phosphoproteomics of human skeletal muscle identify REPS1 as a regulator of muscle glucose uptake
Source: Cell Rep Med. 2025 Jun 6;6(6):102163. doi: 10.1016/j.xcrm.2025.102163 (PMC12208332; doi:10.1016/j.xcrm.2025.102163)
Supplement: Document S1. Figures S1–S5 [file mmc1.pdf]

Cell Reports Medicine, Volume *6*

Supplemental information

Insulin- and exercise-induced phosphoproteomics of human skeletal muscle identify REPS1 as a regulator of muscle glucose uptake

Jeppe Kjærgaard, Cecilie B. Lindqvist, Júlia Prats Quesada, Søren Jessen, Farina Schlabs, Amy M. Ehrlich, Caio Y. Yonamine, Mario García-Ureña, Johann H. Schmalbruch, Lewin Small, Martin Thomassen, Anders Krogh Lemminger, Kasper Eibye, Alba Gonzalez-Franquesa, Jacob V. Stidsen, Kurt Højlund, Juleen R. Zierath, Tuomas O. Kilpeläinen, Jens Bangsbo, Jonas T. Treebak, Morten Hostrup, and Atul S. Deshmukh

Supplemental information

Insulin and Exercise-induced Phosphoproteomics of Human Skeletal Muscle Identify REPS1 as a Regulator of Muscle Glucose Uptake

Jeppe Kjærgaard, Cecilie B. Lindqvist, Júlia Prats Quesada, Søren Jessen, Farina Schlabs, Amy M. Ehrlich, Caio Y. Yonamine, Mario García-Ureña, Johann H. Schmalbruch, Lewin Small, Martin Thomassen, Anders Krogh Lemminger, Kasper Eibye, Alba Gonzalez-Franquesa, Jacob V. Stidsen, Kurt Højlund, Juleen R. Zierath, Tuomas O. Kilpeläinen, Jens Bangsbo, Jonas T. Treebak, Morten Hostrup, Atul S. Deshmukh

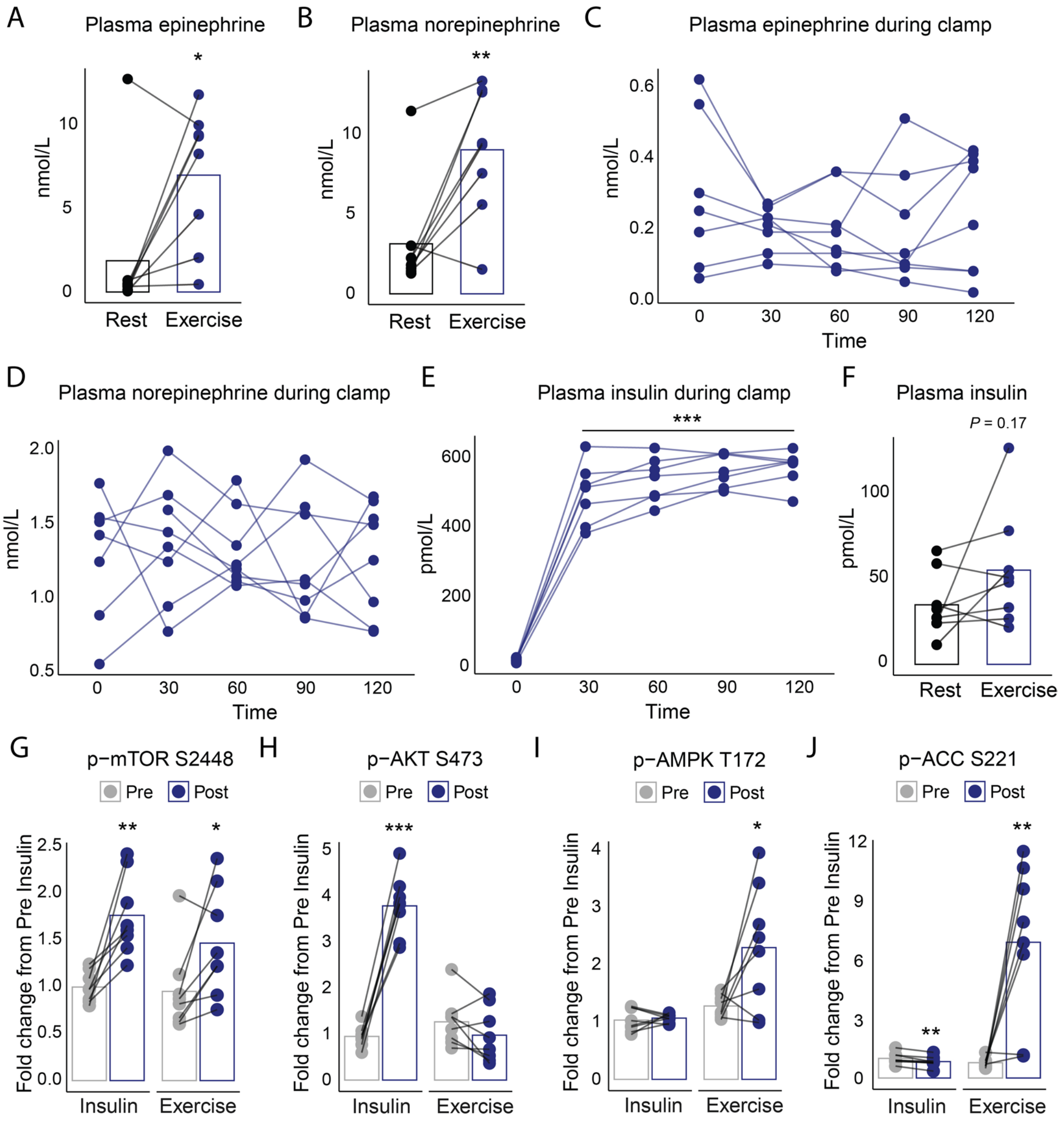

Figure S1. Phosphoproteomic Signature of Insulin and Exercise Signaling in Human Skeletal Muscle. Levels of plasma epinephrine (A), norepinephrine (B) in response to the acute bout of exercise and during the insulin clamp (C-D). Levels of plasma insulin during the insulin clamp (E) and in response to the acute bout of exercise (F). Quantified western blot analysis of p-mTOR S2448 (A), p-AKT S473 (B), p-AMPK T172 (C) and p-ACC S221 (D) in human skeletal muscle pre/post an hyperinsulinemic euglycemic clamp (2hr) and pre/post 10 minutes of high intensity cycling exercise. Paired-Samples t-test was used to analyze mean differences within each experiment. All bars represent the mean. *P* < 0.05 = *, *P* < 0.01 = **, *P* < 0.001 = ***. n=7- 8 for all experiments.

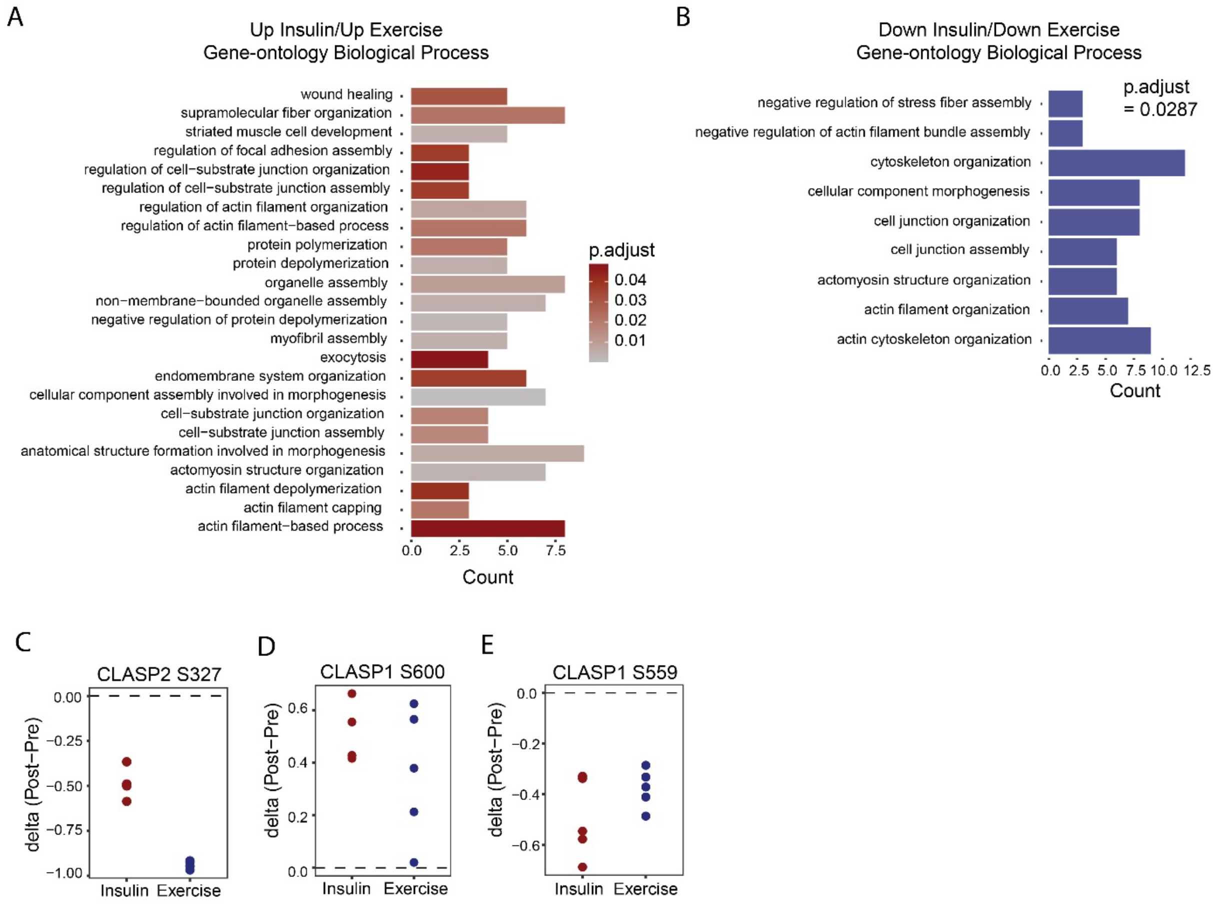

Figure S2 - Shared and Distinct Features of Insulin and Exercise Signaling. Gene Ontology (GO) overrepresentation test was used to assess enrichment for Biological Processes within phosphoproteins being phosphorylated (A) or dephosphorylated (B) by both insulin/exercise. The whole phosphoprotein proteome was used as background. Only BP-terms with a Benjamini Hochberg-adjusted *P* value below 5% is shown. Representation of individual fold changes for CLASP2 S327 (C), CLASP1 S600 (D) and CLASP1 S559 (E) in response to insulin stimulation and an acute bout of exercise by LC-MS/MS.

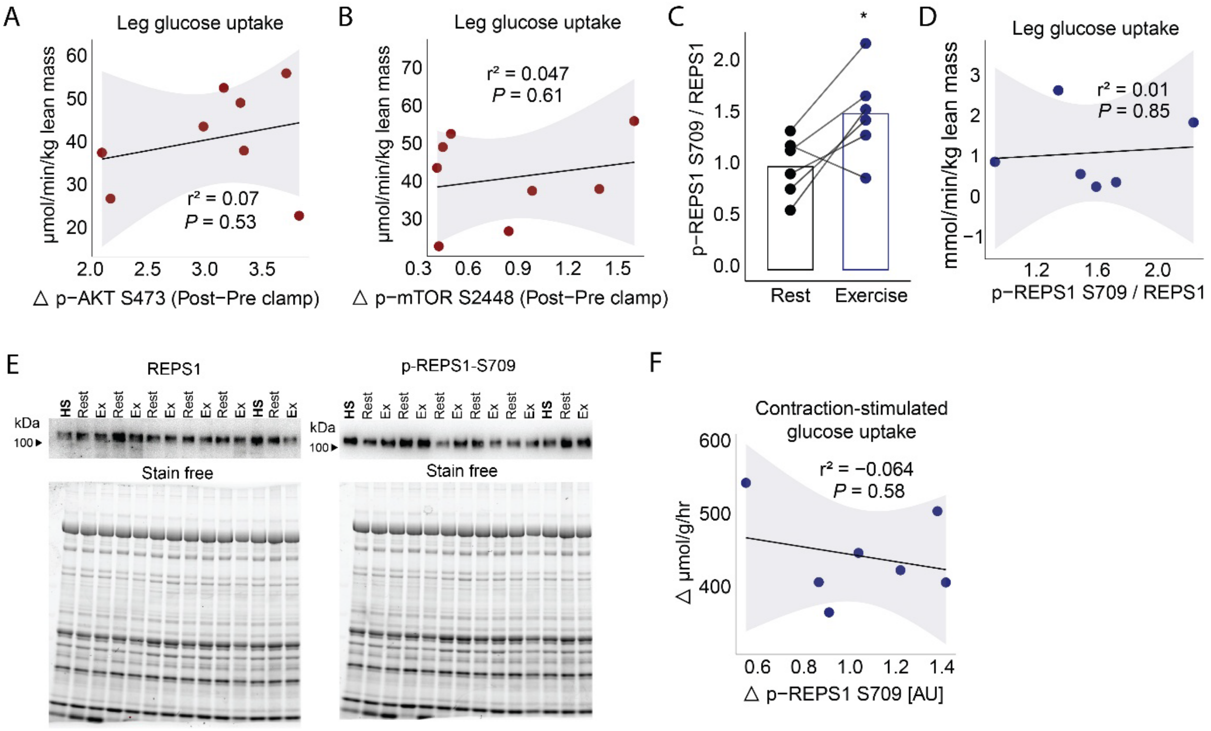

Figure S3. The Insulin and Exercise-responsive Protein, REPS1, is a Critical Regulator of Skeletal Muscle Glucose Uptake. Pearson's correlation analysis of leg steady-state glucose uptake with delta (Post-Pre) p-AKT S473 (A) and p-mTOR S2448 levels (B) measured by western blot (n=8). Quantified western blot of p-REPS1 S709 normalized to total protein at rest and in response to a bout of one-legged exercise (n=6) as mean difference (C) and Pearson's correlation analysis of pREPS1/REPS1 and glucose uptake (D). Representative western blot images (E). Pearson's correlation analysis of delta (Contracted-Rested) glucose uptake and REPS1 S709 phosphorylation in TA mouse muscle (n=7) (D). All bars represent the mean. $P < 0.05$ = *.

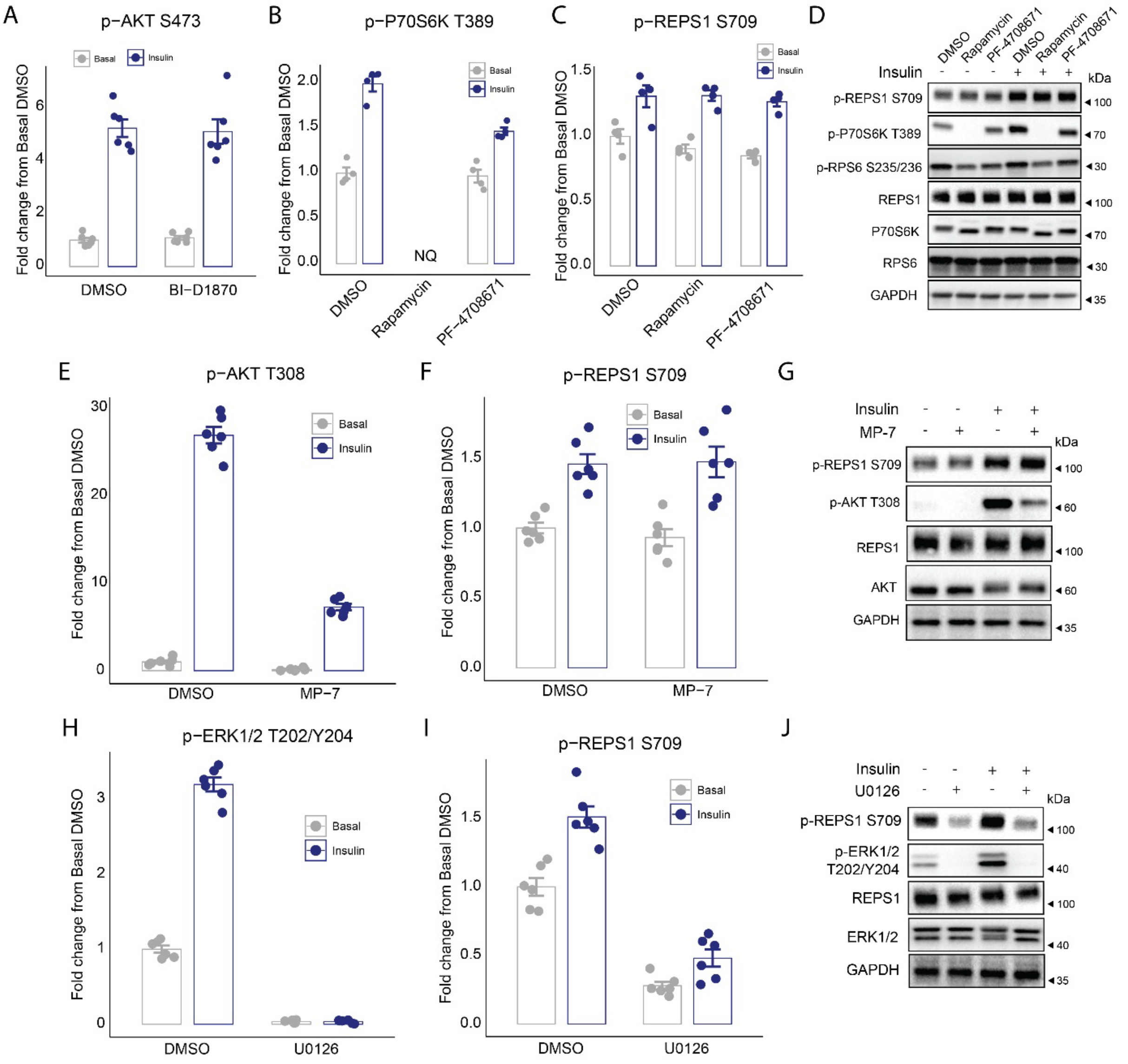

Figure S4. RSK is an Upstream Kinase of REPS1 S709 and is Associated with Vesicle-Sorting Proteins in Skeletal Muscle. Quantified western blot of C2C12 myotubes pretreated with DMSO or 10 μM BI-D1870 for 20 minutes before 10 minutes of 100 nM insulin stimulation (n=6) (A). Quantified western blot of C2C12 myotubes pretreated with DMSO, Rapamycin (100 nM) or PF-4708671 (10 μM) for 20 minutes before 10 minutes of 100 nM insulin stimulation (n=4) (B-C). Representative western blot images for the result presented in B-C (D). Quantified western blot of C2C12 myotubes pretreated with 10 μM MP-7 for 20 minutes followed by 10 minutes of 100 nM insulin stimulation (n=6) (E-F). Representative western blot images for the results presented in E-F (G). Quantified western blot of C2C12 myotubes pretreated DMSO or 10 μM U0126 for 20 minutes before 10 minutes of 100 nM insulin stimulation (n=6) (H-I). Representative western blot images for the results presented in H-I (J). The mean +/- standard error of the mean is displayed.

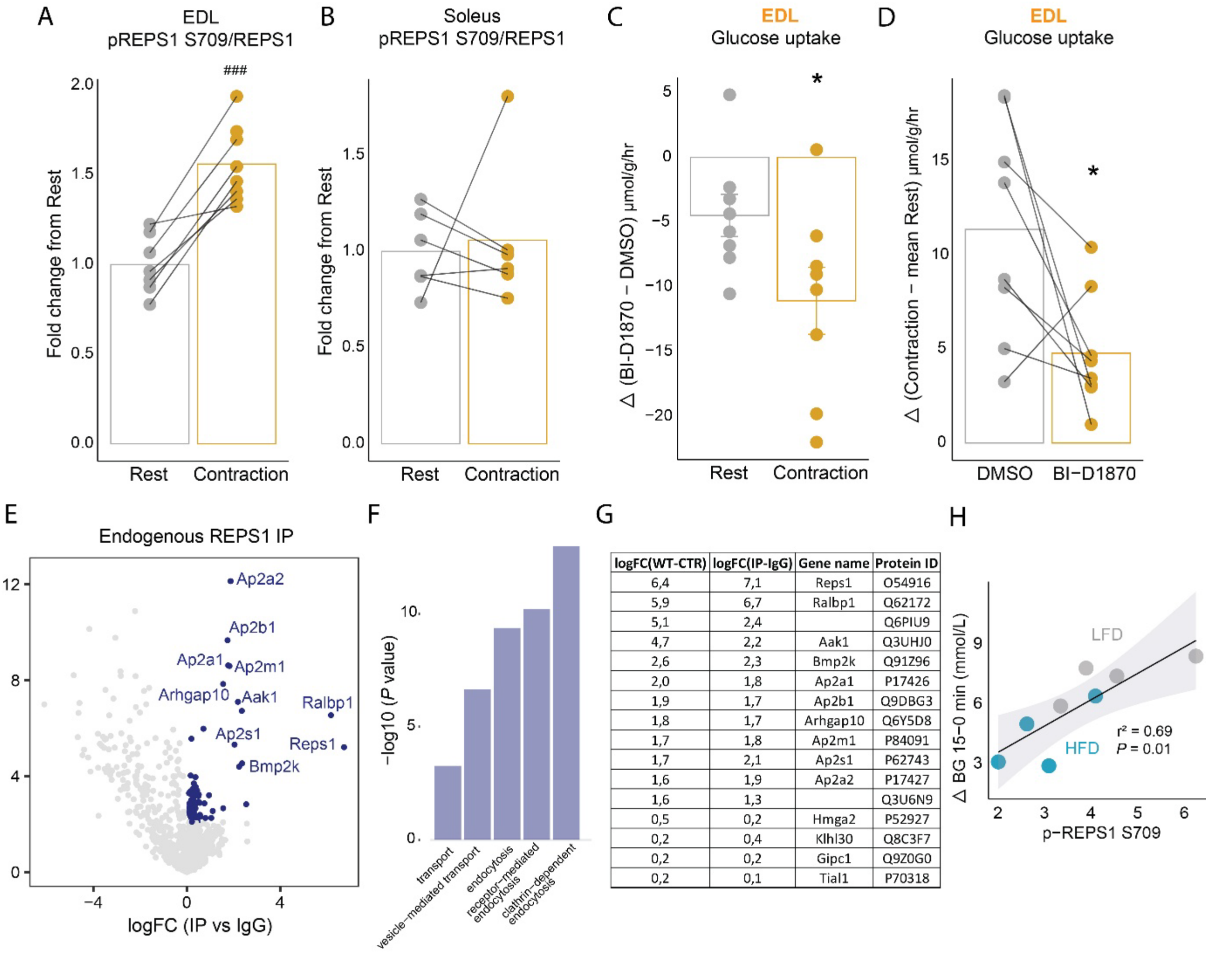

logFC(WT-CTR)	logFC(IP-IgG)	Gene name	Protein ID
6,4	7,1	Reps1	O54916
5,9	6,7	Ralbp1	Q62172
5,1	2,4		Q6PIU9
4,7	2,2	Aak1	Q3UHJ0
2,6	2,3	Bmp2k	Q91Z96
2,0	1,8	Ap2a1	P17426
1,9	1,7	Ap2b1	Q9DBG3
1,8	1,7	Arhgap10	Q6Y5D8
1,7	1,8	Ap2m1	P84091
1,7	2,1	Ap2s1	P62743
1,6	1,9	Ap2a2	P17427
1,6	1,3		Q3U6N9
0,5	0,2	Hmga2	P52927
0,2	0,4	Klhl30	Q8C3F7
0,2	0,2	Gipc1	Q9Z0G0
0,2	0,1	Tial1	P70318

Figure S5. RSK is an Upstream Kinase of REPS1 S709 and is Associated with Vesicle-Sorting Proteins in Skeletal Muscle.

Phosphorylation of REPS1 S709 at rest and in response to contractions in *ex vivo* incubated mouse EDL (n=7) (A) and soleus muscle (n=6) (B). Delta (BI-D1870 – DMSO) glucose uptake between rested and contracted EDL muscle (n=8) (C). Delta (Contraction – Rest) glucose uptake in DMSO- and BI-D1870-treated EDL muscle (n=8) (D). Endogenous IP of REPS1 vs IgG control IP (E). Differentially enriched proteins are highlighted in dark blue (two-sample t-test, FDR < 5%). GO-enrichment analysis of significant interactors found in both pull-down and endogenous IP experiments (F). The whole interactome was used as background. Table showing significant interactors (< 5% FDR) in Flag- and endogenous-IP experiments with log2 fold changes (G). Pearson's correlation analysis of insulin-lowering (delta 15-0 min) blood glucose (BG) and REPS1 S709 phosphorylation in insulin-stimulated quadriceps skeletal muscle from four LFD and four HFD fed mice (A). Data in A, B, and D were analyzed by a two-sided paired sample t-test. Data in C were analyzed by a two-sided unpaired sample t-test. In paired analyses, bars represent the mean. In unpaired analyses, the mean +/- standard error of the mean is displayed. $P < 0.05$ = * effect of inhibitor. $P < 0.001$ = ### effect of contractions.
